# Post‐Translational Modifications in Cilia and Ciliopathies

**DOI:** 10.1002/advs.202416562

**Published:** 2025-05-28

**Authors:** Jie Ran, Jun Zhou

**Affiliations:** ^1^ Center for Cell Structure and Function Shandong Provincial Key Laboratory of Animal Resistance Biology College of Life Sciences Shandong Normal University Jinan 250014 China; ^2^ Department of Genetics and Cell Biology College of Life Sciences State Key Laboratory of Medicinal Chemical Biology Nankai University Tianjin 300071 China

**Keywords:** cilium, ciliopathy, post‐translational modification, signaling, therapy

## Abstract

Cilia are microtubule‐based organelles that extend from the surface of most vertebrate cells, and they play important roles in diverse cellular processes during embryonic development and tissue homeostasis. Mutations in ciliary proteins are associated with a wide range of human diseases, collectively referred to as ciliopathies. The past decades have witnessed significant advances in the identification of post‐translational modifications (PTMs) in ciliary proteins, as well as the enzymes responsible for the PTMs. For example, acetylation of α‐tubulin at lysine 40 is essential for ciliary assembly and maintenance, while ubiquitination of centrosomal proteins, such as pericentriolar material 1, regulates ciliary disassembly. In addition, accumulating evidence has shown that PTMs are essential for modulating ciliary structure and function, and that dysregulation of these modifications leads to the development of ciliopathies. In this review, current knowledge of PTMs in ciliary proteins is summarized, and their roles in regulating ciliary formation, homeostasis, and signaling are highlighted. The contribution of aberrant ciliary PTMs to ciliopathies is also discussed, along with the potential of targeting PTMs for ciliopathy treatment, including pharmacological modulation of PTM‐related enzymes or substrates, which may provide new avenues for therapeutic intervention in ciliopathies.

## Graphical abstract

This review synthesizes current understanding of post‐translational modifications (PTMs) in ciliary proteins and emphasizes their roles in ciliary formation, homeostasis, and signaling. This review also discusses the implication of PTM dysregulation in ciliopathies and explores therapeutic strategies targeting PTM‐modifying enzymes. This review highlights PTMs as pivotal regulators of cilia and suggests PTMs as targets for ciliopathy management.

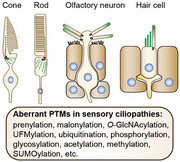

## 1 Introduction

Cilia are evolutionarily conserved organelles characterized by a microtubule‐based structure, known as the axoneme. The axoneme is surrounded by the ciliary membrane and originates from the basal body, a structure derived from the mother centriole.^[^ ^1^ ^]^ Cilia play a critical role in embryonic development, including organ morphogenesis and the establishment of the left‐right asymmetry. In adults, they are essential for maintaining tissue homeostasis, such as contributing to the preservation of photoreceptor integrity and retinal homeostasis, as well as supporting the homeostasis of metabolic and reproductive systems. Disruptions in ciliary structure and function lead to various pathologies known as ciliopathies, which are associated with a wide range of clinical manifestations, such as polycystic kidney disease, neurodegenerative disorders, ocular diseases, obesity, and cancer.^[^ ^2^, ^3^ ^]^

Mechanistic studies have revealed that ciliary abnormalities are often caused by abnormal expression or mislocalization of ciliary proteins, including those that constitute the structural components of cilia and those involved in maintaining ciliary homeostasis, such as ciliary assembly, disassembly, and length control.^[^ ^4^ ^]^ These alterations in ciliary proteins are largely regulated by post‐translational modifications (PTMs), which significantly enhance the complexity of the proteome and are crucial for maintaining tissue homeostasis.^[^ ^5^ ^]^ Examples of PTMs involved in ciliary regulation include acetylation, glycylation, palmitoylation, and the recently identified ubiquitin‐fold modifier 1 (UFM1) modification (UFMylation). These modifications play crucial roles in maintaining ciliary homeostasis and contribute to the pathogenesis of ciliopathies by modifying ciliary proteins.

Several PTMs, including glutamylation, glycylation, acetylation, and detyrosination, have been identified in the microtubules of ciliary axonemes and basal bodies.^[^ ^6^ ^]^ These PTMs are essential for regulating ciliary assembly, disassembly, maintenance, and motility. In addition to the PTMs that occur in the microtubules of the axoneme and basal body, other key structural proteins of ciliary components and regulatory proteins involved in ciliogenesis are also subject to PTMs. For example, palmitoylation of ADP‐ribosylation factor‐like GTPase 13B (ARL13B), a ciliary membrane protein, is essential for protein trafficking and ciliary elongation.^[^ ^7^ ^]^ Similarly, phosphorylation of kinesin family member 3A (KIF3A), a subunit of the kinesin‐2 complex responsible for anterograde transport within cilia, is critical for proper ciliogenesis.^[^ ^8^ ^]^

Accumulating evidence has shown that dysfunction in these PTMs can lead to perturbations in tissue homeostasis, contributing to human diseases.^[^ ^9^ ^]^ For example, the precise regulation of tubulin glutamylation and glycylation in cilia is crucial for sperm development and function. Abnormalities in these PTMs can result in male infertility by disrupting the beating of sperm flagella.^[^ ^10^ ^]^ Furthermore, the interplay between the phosphorylation and ubiquitination of histone deacetylase 6 (HDAC6), a key regulator of ciliary disassembly, has been linked to retinopathy of prematurity through its role in maintaining the integrity of the photoreceptor cilium.^[^ ^11^ ^]^ In this review, we discuss the current understanding of PTMs in the regulation of ciliary structure and function and delve into the intricate connections between PTM abnormalities and human diseases.

## 2 Regulation of Ciliary Structure and Function by PTMs

Cilia are antenna‐like protrusions composed of four main parts: the basal body, transition zone, axoneme, and ciliary membrane. The basal body, situated at the base of the cilium, consists of nine triplet microtubules. During the G0 phase of the cell cycle, the basal body, which originates from the mother centriole, migrates to the cell cortex, where it anchors to the plasma membrane via distal appendages and interacts with microtubules through subdistal appendages. The basal body is connected to the transition zone, a region that functions as a selective barrier, regulating the passage of soluble and membrane‐bound proteins between the cilium and the cell body. The axoneme, extending from the basal body, serves as a microtubule‐based scaffold crucial for ciliary structure and function.^[^ ^12^ ^]^

**Figure 1:**
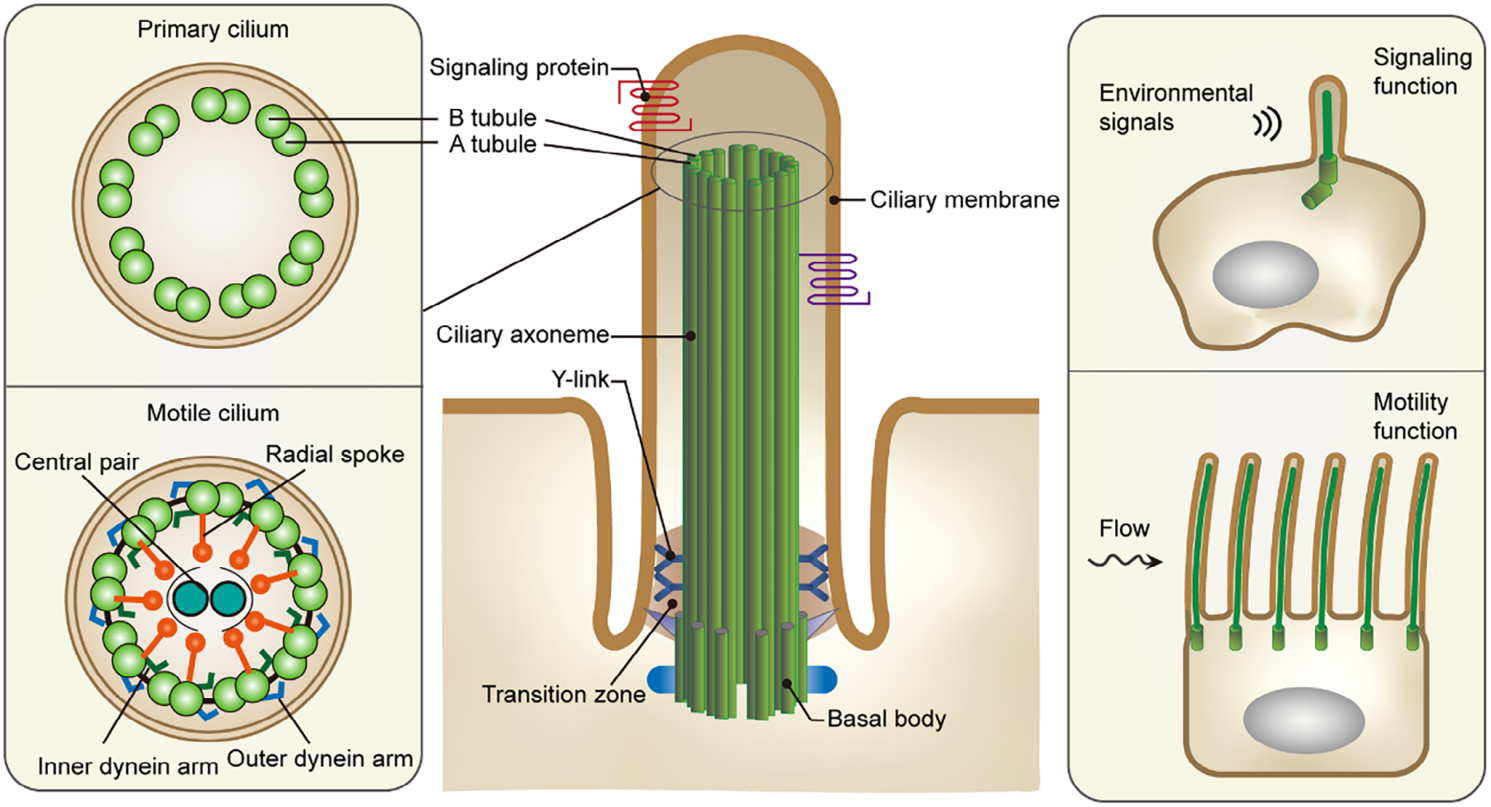
Ciliary structure and function. All cilia originate from basal bodies, which typically comprise triplet microtubules and subdistal and distal appendages. The transition zone is connected to the ciliary membrane via Y‐shaped structures. The axoneme functions as the ciliary backbone and is composed of doublet microtubules. Cilia are traditionally classified into two categories, primary and motile cilia. A single primary cilium is found on the apical surface of most mammalian cells. Primary cilia serve as sensory organelles, capable of detecting environmental signals. In contrast, motile cilia are usually present in large numbers on the cell surfaces of epithelial tissues such as the trachea and oviduct. The axonemes of motile cilia often include additional structures, such as the central pair of microtubules, radial spokes, and inner and outer dynein arms, which are essential for ciliary beating. A variety of PTMs have been identified in proteins localized within the different structures of cilia. These PTMs are crucial for regulating ciliary formation, homeostasis, and functions.

Based on the arrangement and composition of axonemal microtubules, cilia can be categorized into two major types: motile (9+2) cilia and nonmotile primary (9+0) cilia. In 9+2 cilia, nine doublet microtubules encircle a central pair of singlet microtubules. These cilia are typically motile, as they possess axonemal dyneins — molecular motors responsible for ciliary beating. In contrast, 9+0 cilia lack the central microtubule pair and usually also lack axonemal dyneins, making them nonmotile. Motile cilia are often found in large numbers on epithelial cells, such as those lining the respiratory tract, whereas nonmotile cilia are generally solitary. The ciliary membrane, which often invaginates at the base to form the ciliary pocket, contains proteins and lipids distinct from those of the plasma membrane, facilitating unique signaling functions (**Figure** **1**). The intricate arrangement of cilia is highly susceptible to damage, primarily due to abnormal expression or mislocalization of ciliary proteins. These processes are tightly regulated by various PTMs. In this section, we discuss PTMs in ciliary proteins and their roles in ciliary formation, homeostasis, and signaling (**Table** **1**).

**Table 1:** Representative PTMs in ciliary compartments: Modifying enzymes and their substrates.

Ciliary Components	PTMs	Enzymes	Substrates
Basal body	Ubiquitination	MIB1, USP9X	PCM1,^[^ ^16^, ^18^ ^]^ CEP131,^[^ ^20^ ^]^ CEP290,^[^ ^16^ ^]^ OFD1^[^ ^21^ ^]^
		CYLD	CEP70,^[^ ^148^ ^]^ CAP350,^[^ ^19^ ^]^ PCM1^[^ ^17^ ^]^
		USP33	CP110^[^ ^23^ ^]^
		HUWE1	TTBK2^[^ ^122^ ^]^
	Phosphorylation	PLK4	PCM1^[^ ^26^ ^]^
		TTBK2	CEP164,^[^ ^28^ ^]^ CEP83,^[^ ^29^ ^]^ MPP9^[^ ^31^ ^]^
		MARK4	ODF2^[^ ^30^ ^]^
		ASK1	HDAC6^[^ ^11^ ^]^
Transition zone	Ubiquitination	VHL, SYVN1	HDAC6,^[^ ^11^ ^]^ KIF11^[^ ^82^ ^]^
	Prenylation	PDEδ	RPGR^[^ ^78^, ^79^ ^]^
	UFMylation	UFL1	KIF11^[^ ^82^ ^]^
	Glutamylation	TTLL5	RPGR^[^ ^80^ ^]^
Axoneme	Acetylation	αTAT1,^[^ ^39^ ^]^ HDAC6,^[^ ^41^ ^]^ SIRT2^[^ ^40^ ^]^	α/β‐tubulin
	Glutamylation	TTLL1,^[^ ^52^ ^]^ TTLL3,^[^ ^47^ ^]^ TTLL6,^[^ ^47^ ^]^ TTLL8,^[^ ^47^ ^]^ TTLL9,^[^ ^51^ ^]^ TLL11,^[^ ^54^ ^]^ CCP1,^[^ ^54^ ^]^ CCP5,^[^ ^50^ ^]^ CCP6^[^ ^93^ ^]^	
	Detyrosination^[^ ^59^ ^]^	SVBP/Vasohibin	
	Glycylation	TTLL3,^[^ ^47^ ^]^ TTLL6,^[^ ^47^ ^]^ TTLL8,^[^ ^53^ ^]^ TTLL10^[^ ^149^ ^]^	
	Δ2‐tubulin^[^ ^58^ ^]^	Not elucidated	
Ciliary membrane	SUMOylation	dPIAS, SENP2	Smoothened,^[^ ^87^ ^]^ AC3^[^ ^88^ ^]^
	Phosphorylation	PKA,^[^ ^84^ ^]^ CK1,^[^ ^83^ ^]^ GRK2^[^ ^85^ ^]^	Smoothened
	Palmitoylation	CPT1	ARL13B^[^ ^7^ ^]^
IFT system	Phosphorylation	MAK, ICK	IFT88^[^ ^65^ ^]^ KIF3A,^[^ ^8^, ^65^ ^]^ IFT57^[^ ^65^ ^]^
	Ubiquitination	XIAP	IFT88^[^ ^92^ ^]^
	Glutamylation^[^ ^133^ ^]^	TTLL11, CCP1	Not elucidated

### 2.1 PTMs in Basal Body Proteins for Ciliary Formation

**Figure 2:**
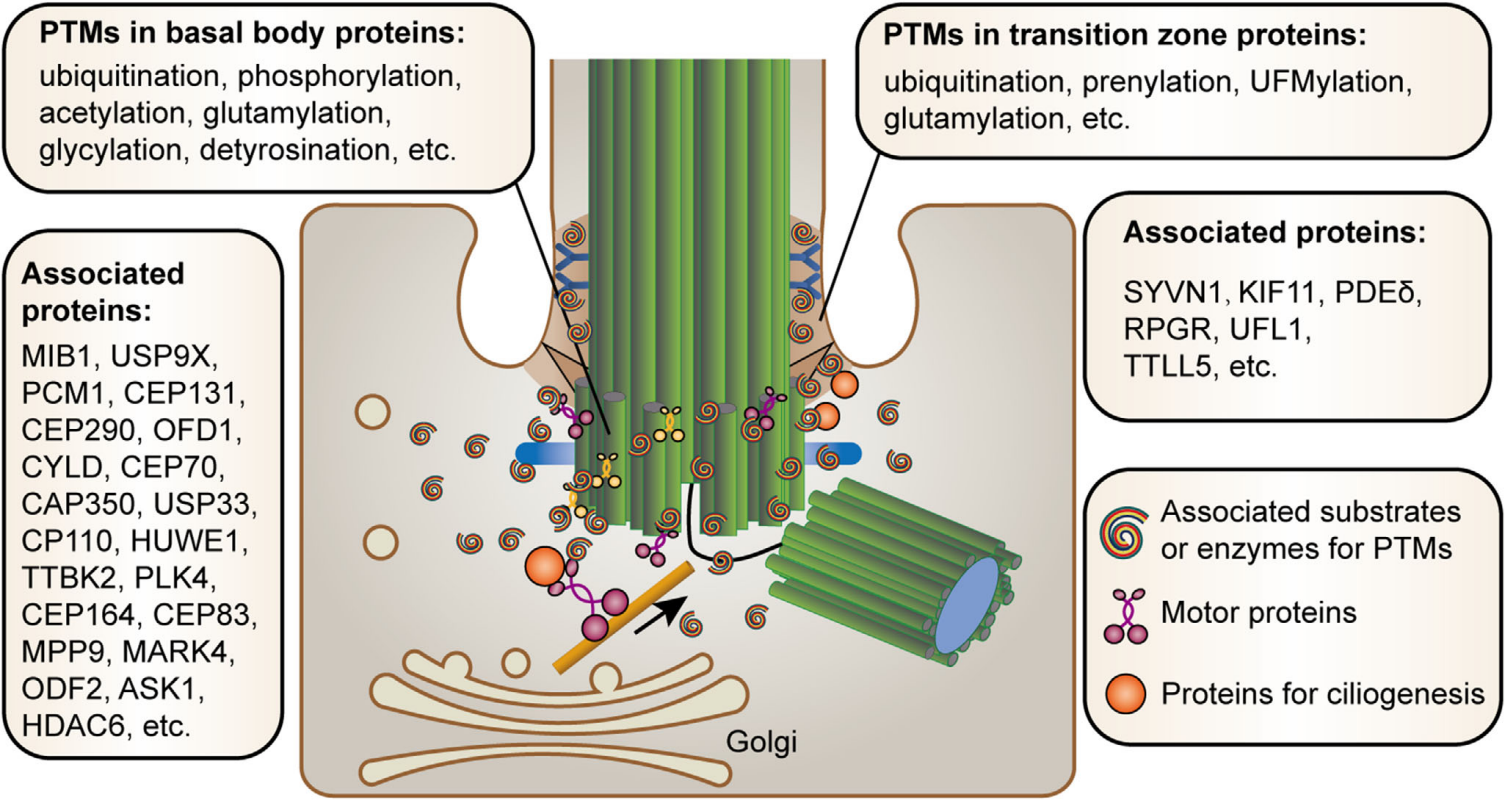
PTMs in ciliary basal body and transition zone proteins during early stages of ciliogenesis. The initiation of ciliogenesis originates from the transformation of the mother centriole into a basal body, a process tightly modulated by key protein components associated with the mother centriole. These include proteins of the distal and subdistal appendages, such as the subdistal appendages member ODF2, the distal appendages members CEP164 and TTBK2, and the centriolar satellite proteins OFD1 and PCM1, along with the removal of the negative regulator CP110 from the mother centriole. These regulatory proteins undergo various PTMs, which play crucial roles in orchestrating the initiation of ciliogenesis. Once the basal body matures, the transition zone (known as the connecting cilium in photoreceptor cells) forms and organizes a diffusion barrier for ciliary formation. This process is also regulated by PTMs. For example, prenylation of the RPGR protein is crucial for its proper localization in the transition zone and the trafficking of ciliary membrane proteins. Additionally, key components in the connecting cilium of photoreceptor cells, such as KIF11, are regulated by coordinated UFMylation and ubiquitination to maintain photoreceptor integrity.

The basal body is a highly conserved, microtubule‐based, membrane‐less structure that plays a crucial role in the nucleation of the ciliary axoneme. Structurally, it consists of nine triplets of microtubules arranged in a ninefold symmetry, forming a cylindrical shape, and is surrounded by the pericentriolar material (PCM). The basal body is also equipped with subdistal appendages, which anchor microtubules, and distal appendages, which mediate the docking of the basal body to the plasma membrane during ciliogenesis. At the PCM periphery, granular structures known as centriolar satellites are composed of the core scaffold protein PCM1 and other key proteins, including centrosomal protein 131 (CEP131), CEP290, oral‐facial‐digital syndrome 1 protein (OFD1), Bardet‐Biedl syndrome 4 (BBS4), ninein, and mind bomb1 (MIB1). These basal body proteins undergo various PTMs, which are critical for basal body maintenance and ciliary formation (**Figure** **2**).

Ubiquitination is a common PTM that is involved in ciliary biology.^[^ ^13^ ^]^ Early proteomic analyses of isolated centrosomes and cilia revealed numerous proteins, including E3 ubiquitin ligases and deubiquitinases, that target specific substrates during various stages of ciliary assembly and disassembly pathways.^[^ ^14^, ^15^ ^]^ For example, MIB1‐mediated polyubiquitination of centriolar satellite proteins, such as PCM1, CEP131, and OFD1, reduces ciliogenesis.^[^ ^16^ ^]^ In contrast, deubiquitinases such as ubiquitin‐specific peptidase 9 X‐linked (USP9X) and cylindromatosis (CYLD) counteract this process, promoting ciliogenesis.^[^ ^17^, ^18^, ^19^, ^20^, ^21^ ^]^ Additionally, other enzymes of ubiquitination and deubiquitination, along with ciliary proteins localized to the basal body, play essential roles in ciliary formation and have been implicated in a variety of human diseases. For example, the ubiquitination of the centrosomal kinase tau tubulin kinase 2 (TTBK2) by HUWE1 (HECT, UBA, and WWE domain‐containing protein 1) promotes its degradation, leading to the disassembly of primary cilia and suggesting a potential target for medulloblastoma therapy.^[^ ^22^ ^]^ Furthermore, CP110 levels are regulated by ubiquitination via the SCF‐cyclin F and EDD‐DDB1‐VprBP complexes, along with the deubiquitinase USP33, influencing the conversion of the mother centriole to the basal body and offering a strategy to inhibit tumorigenesis linked to centrosome amplification.^[^ ^23^, ^24^, ^25^ ^]^

Phosphorylation is another crucial PTM in basal body proteins. PCM1 phosphorylation by polo‐like kinase 4 (PLK4) at serine 372 is essential for its dimerization and scaffolding activity, which facilitates the integrity of centriolar satellites and ciliogenesis.^[^ ^26^ ^]^ TTBK2, a serine/threonine kinase, is recruited to distal appendages to initiate ciliogenesis by removing CP110 from the mother centriole.^[^ ^27^ ^]^ It also phosphorylates distal appendage proteins, such as CEP164 and CEP83.^[^ ^28^, ^29^ ^]^ Microtubule‐associated protein/MT affinity regulating kinase 4 (MARK4), another centrosomal kinase, modulates the phosphorylation and centrosomal localization of ODF2, influencing ciliary initiation and assembly.^[^ ^30^ ^]^ A crosstalk between phosphorylation and ubiquitination is frequently observed in ciliary regulation. For example, TTBK2 phosphorylates M‐phase phosphoprotein 9 (MPP9), promoting its ubiquitination and degradation, which facilitates the removal of CP110 and CEP97 from the mother centriole, enabling ciliary initiation.^[^ ^31^ ^]^ Similarly, ASK1‐mediated phosphorylation stabilizes HDAC6 by preventing its ubiquitination, promoting ciliary disassembly in retinopathy of prematurity.^[^ ^11^ ^]^

Ciliary microtubules are also subject to several PTMs, including acetylation, polyglutamylation, glycylation, and detyrosination, which are frequently observed in the ciliary basal body. Notably, polyglutamylation serves as an early hallmark of centriole/basal body assembly, underscoring its role in maintaining centriolar architecture and stability.^[^ ^32^ ^]^ A recent study has identified C11ORF49/CSTPP1 as a novel regulator of the tubulin polyglutamylase complex (TPGC), which modulates the polyglutamylation of microtubules. Loss of C11ORF49/CSTPP1 disrupts TPGC assembly and stability, leading to aberrant microtubule assembly and ciliogenesis.^[^ ^33^ ^]^ Additionally, glycylation of basal body‐appendage microtubules has been shown to promote their cortical attachment, ensuring proper basal body positioning and organization of ciliary arrays.^[^ ^34^ ^]^ These PTMs are not restricted to the basal body but are also present on the ciliary axoneme, where they collaboratively regulate microtubule stability and ciliary function. Thus, these microtubule‐specific PTMs provide a conserved regulatory mechanism linking the basal body and axonemal functions in the context of ciliogenesis and ciliary maintenance.

### 2.2 PTMs in Axonemal Proteins for Ciliary Maintenance

**Figure 3:**
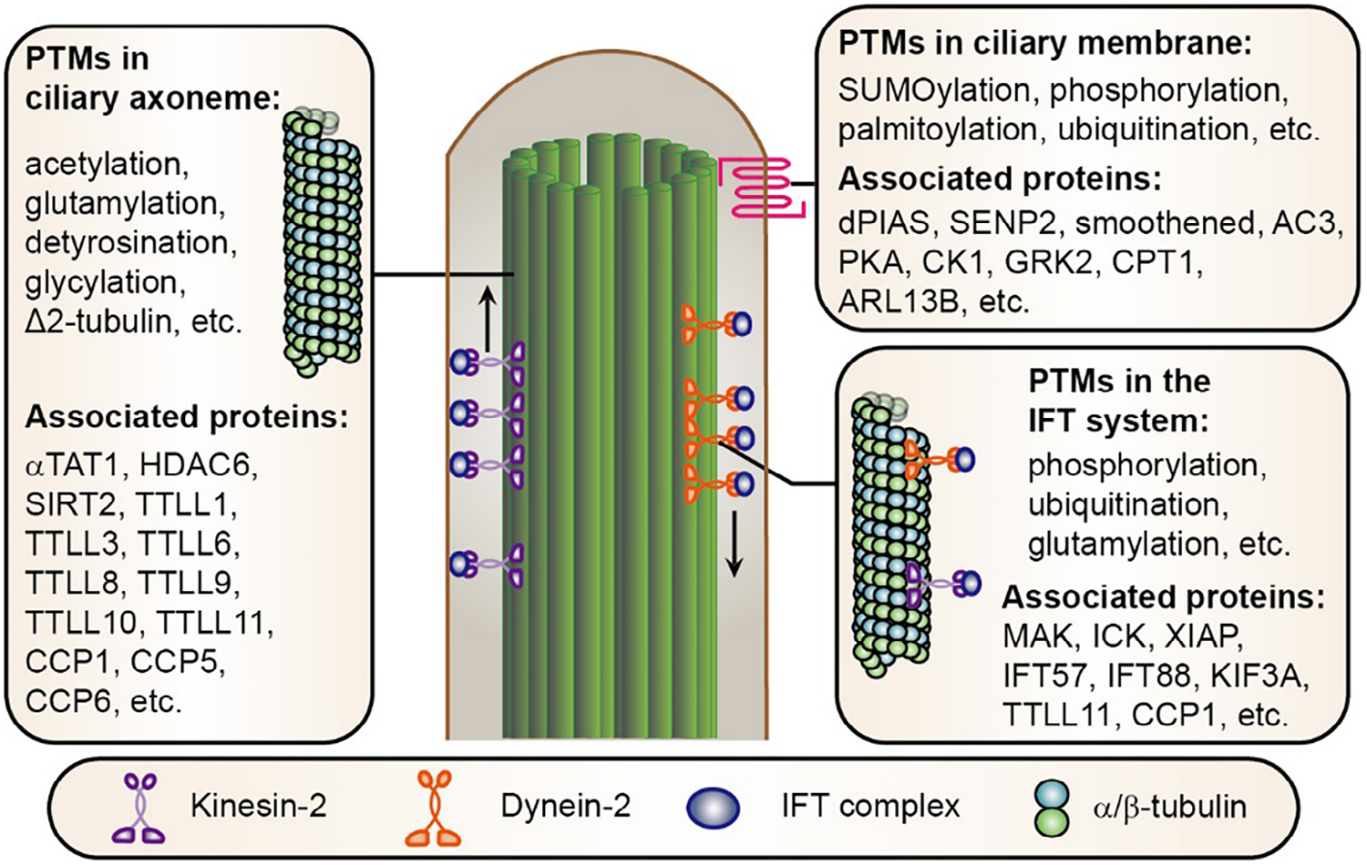
PTMs in ciliary elongation. The microtubule‐based ciliary axoneme is a pivotal structural component of the cilium and extends from the basal body and transition zone. Similar to cytoplasmic microtubules, axonemal microtubules exhibit dynamic behavior and undergo processes such as assembly and disassembly. These processes are intricately modulated by various PTMs. Continued elongation of the cilium requires the import and transport of ciliary proteins to the tip by the IFT system.  IFT is a bidirectional transport system driven by motor proteins, including kinesins for anterograde transport and dyneins for retrograde transport. This transport machinery is also regulated by PTMs such as phosphorylation, ubiquitination, and glutamylation, ensuring the precise coordination of protein transport and ciliary elongation.

The ciliary axoneme is composed of nine parallel doublet microtubules known as outer doublets, which elongate from the basal body. The tubulin of the outer doublets is subjected to several conserved PTMs, including acetylation, glutamylation, and glycylation, which are crucial for ciliary maintenance and function (**Figure** **3**). Dysregulation of these PTMs is linked to various diseases, such as male infertility, cancer, retinal degeneration, respiratory disorders, neural disorders, blood disorders, and cardiomyopathy.

Acetylation of α‐tubulin at lysine 40 (K40) is a highly conserved PTM and the only known modification located inside the microtubule lumen. This modification was initially discovered in the cilia of *Chlamydomonas reinhardtii*.^[^ ^35^, ^36^ ^]^ The enzyme responsible for generating acetylated K40 is α‐tubulin acetyltransferase 1 (αTAT1)/MEC‐17.^[^ ^37^, ^38^ ^]^ Studies have shown that αTAT1 is universally and exclusively conserved in ciliated organisms and is essential for the acetylation of axonemal microtubules and ciliary assembly.^[^ ^39^ ^]^ Additionally, αTAT1 depletion leads to impaired sperm motility and male infertility in mice and reduces touch sensitivity in *Caenorhabditis elegans*.^[^ ^38^, ^39^ ^]^ In contrast, the two K40 deacetylases, HDAC6 and sirtuin 2 (SIRT2), promote ciliary disassembly.^[^ ^40^, ^41^ ^]^ Furthermore, various studies have demonstrated that HDAC6‐mediated ciliary disassembly plays a crucial role in maintaining tissue homeostasis and in various ciliopathies such as respiratory disorders, retinal diseases, male infertility, and cancer. This suggests a potential for targeting HDAC6‐mediated tubulin deacetylation in the treatment of these diseases.^[^ ^42^, ^43^, ^44^, ^45^, ^46^ ^]^

Glutamylation and glycylation are two related PTMs that form as peptide side chains on the C‐terminal tail of tubulin. These two tubulin PTMs are referred to as “polymodifications” due to their polymeric nature. Glutamylation is abundant on long‐lived microtubules, including those found in ciliary axonemes and basal bodies, as well as spindle microtubules, whereas glycylation is restricted to the axonemes and basal bodies of cilia. Polymodifications are catalyzed by TTL‐like proteins (TTLLs), whereas deglutamylation is catalyzed by cytosolic carboxypeptidases (CCPs). Tubulin glutamylation is a crucial regulator of ciliary beating, and the dysfunction of this modification leads to aberrations in motile cilia, such as sperm flagella and cilia of the trachea and ependyma, resulting in male infertility, impaired mucous flow, and neurodegeneration.^[^ ^47^, ^48^, ^49^, ^50^, ^51^, ^52^ ^]^ Similarly, dysregulation of glycylation affects sperm motility and reduces male fertility.^[^ ^10^ ^]^ Additionally, recent studies have shown that nonmotile ciliary functions are also regulated by polymodifications, particularly in the cilia of photoreceptors.^[^ ^53^, ^54^ ^]^ Dysregulation of these two PTMs can result in shortened photoreceptor cilia and progressive retinal degeneration.^[^ ^55^ ^]^ Interestingly, dysregulation of glutamylation has been linked to Joubert syndrome (JBTS), a classic ciliopathy, as well as angiogenesis mediated by endothelial cell cilia, revealing new functions for this modification.^[^ ^56^, ^57^ ^]^ Other tubulin PTMs, such as detyrosination and Δ2‐tubulin, are also enriched in the axonemes of cilia or flagella.^[^ ^58^ ^]^ In cilia, detyrosinated tubulin accumulates on the B‐tubules of outer doublets, increasing the velocity and processivity of kinesin‐2, suggesting that this PTM could stimulate anterograde intraflagellar transport (IFT).^[^ ^59^ ^]^

### 2.3 PTMs in IFT Proteins for Ciliary Length Control

IFT is a complex and highly conserved microtubule‐based transport system that facilitates the movement of ciliary proteins along axonemal microtubules. This bidirectional protein trafficking system comprises the antegrade IFT‐B complex, which is driven by the heterotrimeric kinesin‐2 KIF3A/KIF3B, and the retrograde IFT‐A complex, mediated by the cytoplasmic dynein‐2 motor.^[^ ^60^, ^61^ ^]^ In vertebrates, IFT is thought to be crucial for ciliary length control and, consequently, embryonic development.^[^ ^62^ ^]^ For example, IFT complex B proteins, including IFT88, IFT54, and IFT20, modulate the Hippo pathway effector YAP1 through modulation of ciliary length during embryonic development, which is essential for restricting the formation of the proepicardium and myocardium during cardiogenesis.^[^ ^63^ ^]^ Additionally, depletion of IFT46, a core component of the IFT‐B complex, leads to shortened and abnormal cilia in zebrafish and mice, resulting in developmental defects in the brain, neural tube, and heart.^[^ ^64^ ^]^ Notably, *Ift46* knockout mice display randomization of embryonic heart looping, a hallmark of defective left‐right axis patterning.^[^ ^64^ ^]^ Many mutations in mouse IFT components lead to skeletal ciliopathies and are linked to other ciliopathies, including retinitis pigmentosa and nephronophthisis.

This system is regulated by several PTMs, particularly phosphorylation (Figure 3). Two serine/threonine kinases, male germ cell‐associated kinase (MAK) and intestinal cell kinase (ICK), are proposed to serve as regulators of IFT turnaround at the ciliary tip and ciliary length.^[^ ^8^, ^65^ ^]^ MAK localizes to the ciliary axoneme in photoreceptors and is essential for ciliary length control by modulating the accumulation of the ciliary transport proteins IFT88 and IFT57 at the ciliary tip.^[^ ^65^ ^]^ In addition, *Mak* depletion or mutation causes retinal degenerative diseases in both mice and humans.^[^ ^66^, ^67^ ^]^ In contrast, ICK primarily localizes to the ciliary tip as a regulator of ciliary transport, promoting ciliary assembly by phosphorylating KIF3A.^[^ ^8^, ^68^ ^]^ *Ick* knockout mice exhibit neonatal lethality accompanied by developmental abnormalities in multiple organ systems, including the lung, bone, brain, kidney, retina, and ear.^[^ ^69^ ^]^ Furthermore, various *Ick* mutations have been identified in patients with ciliopathies, such as short rib‐polydactyly syndrome, neural disorders, and polydactyly.^[^ ^70^, ^71^, ^72^ ^]^ In addition, a recent study uncovered a crucial role of IFT88 ubiquitination by X‐linked inhibitor of apoptosis (XIAP) in regulating ciliary homeostasis during the pathogenesis of liver fibrosis, suggesting a potential for targeting the XIAP‐IFT88 axis in the treatment of liver fibrosis.^[^ ^73^ ^]^

Moreover, the IFT system is also modulated by polyglutamylation. Polyglutamylation positively regulates IFT and certain microtubule motors, whereas the removal of polyglutamylation in cilia of engineered cell lines impairs anterograde IFT dynamics.^[^ ^59^ ^]^ A recent study using mouse models with increased glutamylation (*Ccp5^−/−^* and *Ccp1^−/−^*) has demonstrated that aberrant glutamylation disrupts the outer segment architecture of photoreceptor cells. This disruption includes the exacerbation of the connecting cilium, loss of the bulge region, and the destabilization of the distal axoneme. Importantly, these models have also revealed a significant reduction in IFT88 protein intensity, further highlighting the essential role of proper glutamylation in regulating IFT protein levels and ensuring the structural integrity of the photoreceptor cilia.^[^ ^74^ ^]^

### 2.4 PTMs in Transition Zone Proteins for Ciliary Gating

The ciliary transition zone, a region between the basal body and the axoneme, serves as a gatekeeper regulating the import and export of proteins in the cilium. This ensures the distinct composition of the cilium, separate from other subcellular compartments.^[^ ^75^ ^]^ This function relies on the proper localization and function of transition zone module proteins, such as Meckel−Gruber syndrome (MKS) proteins, nephronophthisis (NPHP) proteins, CEP290, and retinitis pigmentosa GTPase regulator (RPGR)−interacting protein 1−like protein (RPGRIP1L). These proteins are critical for transition zone formation and have been implicated in various syndromic ciliopathies, including MKS, NPHP, JBTS, oral‐facial‐digital syndrome, and Senior‐Loken syndrome.^[^ ^76^ ^]^

Recent studies have shown that PTMs in these transition zone modulators are crucial for ciliary maintenance and function (Figure 2). For example, prenylation, a post‐translational lipid modification that occurs via the covalent attachment of one or two prenyl groups, modulates protein‐protein interactions and the subcellular localization of proteins.^[^ ^77^ ^]^ This modification is required for the ciliary localization of RPGR, thereby ensuring ciliary trafficking in photoreceptors.^[^ ^78^ ^]^ The RPGR is localized at the connecting cilium, which is analogous to the transition zone of the primary cilium.^[^ ^46^, ^79^ ^]^ Notably, the RPGR function in photoreceptor cilia requires glutamylation by TTLL5, which is a modification essential for proper opsin trafficking and photoreceptor survival.^[^ ^80^ ^]^ Furthermore, proteins localized in the photoreceptor cilia are regulated by other PTMs, such as UFMylation, a ubiquitination‐like modification.^[^ ^81^ ^]^ A recent study has revealed that UFMylation of KIF11, a ciliary protein situated at the connecting cilium of photoreceptors, is crucial for maintaining photoreceptor cilium integrity and retinal homeostasis.^[^ ^82^ ^]^ Additionally, the phosphorylation of HDAC6 enhances its localization to the photoreceptor connecting cilium, promoting ciliary disassembly associated with retinopathy of prematurity.^[^ ^11^ ^]^

### 2.5 PTMs in Ciliary Membrane Proteins for Ciliary Signaling

The ciliary membrane, although connected to the plasma membrane, possesses a unique composition of proteins and lipids that mediate cell signaling. These components dynamically localize to cilia in response to cellular and environmental cues. Notably, these proteins are also regulated by PTMs, which in turn control their ciliary localization and signaling activity (Figure 3). For example, the phosphorylation of smoothened, a seven‐transmembrane protein that is involved in sonic hedgehog signaling, by kinases such as protein kinase A (PKA), casein kinase I (CK1), and G protein‐coupled receptor kinase 2 (GRK2), is crucial for its ciliary localization and active conformation.^[^ ^83^, ^84^, ^85^, ^86^ ^]^ Additionally, SUMOylation, a reversible covalent modification, counteracts smoothened ubiquitination, leading to its accumulation in the ciliary membrane and enhanced Hedgehog signaling.^[^ ^87^ ^]^ Other ciliary membrane proteins, such as adenylyl cyclase 3 (AC3), a ciliary marker associated with odorant receptors, also undergo SUMOylation. This modification regulates AC3 ciliary entry, which is vital for its role in odor detection.^[^ ^88^ ^]^

ARL13B is a small G protein that is mutated in patients with JBTS, a ciliopathy characterized by ciliary dysfunction that leads to neurological and developmental deficiencies.^[^ ^89^ ^]^ ARL13B is highly enriched in the ciliary membrane and is required for ciliary formation and Sonic Hedgehog signaling.^[^ ^90^ ^]^ Studies indicate that palmitoylation, the reversible lipid attachment of a saturated 16‐carbon palmitate to a cysteine side chain, is crucial for the association of ARL13B with the ciliary membrane and its efficient trafficking to cilia.^[^ ^91^ ^]^ Additionally, palmitoylation enhances ARL13B stability and is necessary for ciliary elongation.^[^ ^7^ ^]^ Furthermore, recent research has revealed a significant role for ARL13B palmitoylation in regulating ciliary homeostasis during the pathogenesis of atherosclerosis. Importantly, restoring palmitic acid availability can significantly restore endothelial cell cilia and mitigate the progression of atherosclerosis, suggesting a promising strategy for the prevention and treatment of this condition.^[^ ^92^ ^]^

## 3 Dysregulation of PTMs in Ciliopathies and the Therapeutic Implications

In the past few decades, significant advances have been made in understanding how PTMs in ciliary proteins regulate ciliary structure and function. Manipulating these PTMs in various model organisms has elucidated their physiological roles and potential links to various diseases. Additionally, direct connections between PTMs and ciliopathies have emerged with the discovery of mutations in enzymes responsible for these PTMs. In this section, we discuss how the dysregulation of ciliary PTMs and the associated enzymes might contribute to ciliopathies and how aberrant PTMs could be targeted for ciliopathy treatment.

### 3.1 Perturbation of PTMs in Motile Ciliopathies

The spectrum of ciliopathies is rapidly expanding. Motile ciliopathies range from male subfertility to multisystem disorders such as hydrocephalus, situs inversus, and primary ciliary dyskinesia. Recent studies have highlighted the close link between PTMs and motile ciliopathies. For example, tubulin glycylation plays a significant role in controlling male fertility. Deficiency in the glycylating enzymes, such as TTLL3 and TTLL8, which are responsible for adding the glycine residues to unmodified tubulin, results in subfertility owing to reduced sperm motility and altered flagellar beating patterns.^[^ ^10^ ^]^ Furthermore, disruption in glutamylation not only leads to male infertility but also causes abnormal beating of cilia in the trachea and brain ependyma, contributing to symptoms of primary ciliary dyskinesia and hydrocephalus.^[^ ^93^ ^]^ Although both glycylation and glutamylation affect the beating of motile cilia, their mechanisms of action are distinct. A recent study using immuno‐cryo‐electron tomography, expansion microscopy, and mutation analysis demonstrated that, in motile cilia, tubulin glycylation and polyglutamylation form mutually exclusive protofilament‐specific nanopatterns. This conserved nanopattern of polyglutamylation and glycylation within the axoneme is essential for the proper function of the nexin−dynein regulatory complex and axonemal dyneins, respectively.^[^ ^94^ ^]^

### 3.2 Dysregulation of PTMs in Sensory Ciliopathies

**Figure 4:**
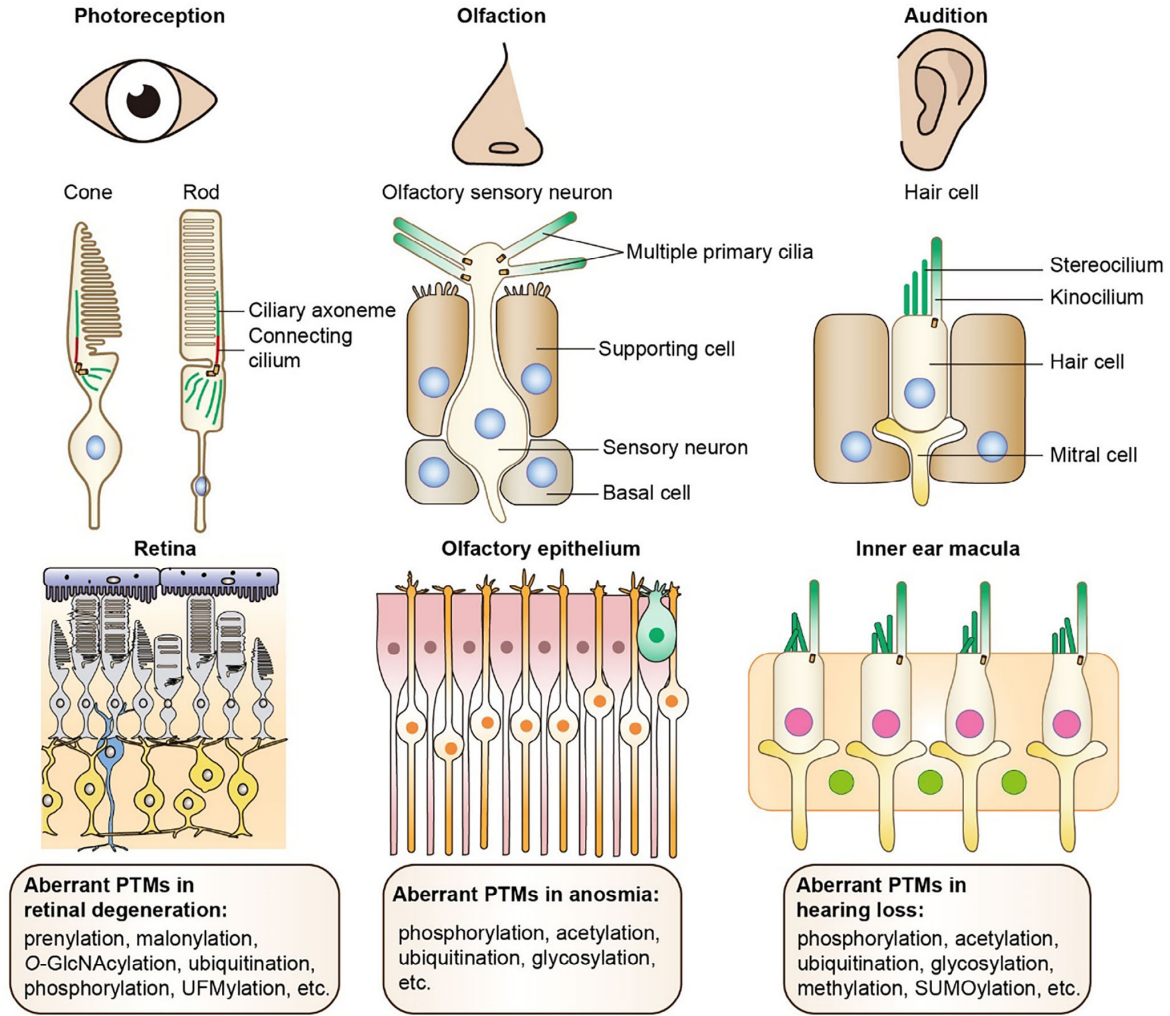
Impaired perception of environmental cues in sensory ciliopathies. The perception of environmental cues is dependent on the sensory systems. As cellular antenna, primary cilia are involved in fundamental biological processes such as photoreception, olfaction, and audition. Sensory ciliopathies, caused by deficiencies in ciliary structure and function, lead to sensory impairments, including retinal degeneration, hearing loss, and anosmia. These sensory ciliary disorders involve the dysregulation of various PTMs. For example, retinal disorders resulting from defects in photoreceptor ciliary proteins are associated with PTMs such as prenylation, malonylation, *O*‐GlcNAcylation, ubiquitination, phosphorylation, and UFMylation. Similarly, PTMs, including phosphorylation, acetylation, ubiquitination, and glycosylation, are linked to both anosmia and hearing loss. Additionally, hearing loss is influenced by methylation and SUMOylation.

Sensory ciliopathies result primarily from defects in nonmotile cilia. Various cell types utilize distinct forms of sensory cilia to perceive external signals, allowing them to perform specific physiological functions such as vision, audition, and olfaction. For example, in the human retina, the photoreceptor outer segment acts as a modified primary cilium where the initial stages of vision occur. In hair cells responsible for hearing, a single microtubule‐based kinocilium and multiple actin‐based stereocilia work together to convert sound frequencies into electrical signals. Similarly, olfactory receptor cells (ORCs) have multiple cilia that extend from the dendritic tip into the extracellular mucus layer to translate chemical information from odor molecules into electrical signals (**Figure** **4**).

Defects in these sensory cilia are associated with sensory ciliopathies such as retinal degeneration, deafness, and anosmia, which are linked to alterations in the PTMs of ciliary proteins (Figure 4). For example, prenylation of RPGR has been implicated in retinal diseases, including retinitis pigmentosa 2 and JBTS.^[^ ^78^, ^95^ ^]^ Recent studies have identified several novel PTMs, such as malonylation, *O*‐GlcNAcylation, and UFMylation, in the pathogenesis of retinal ciliopathies.^[^ ^96^, ^97^, ^98^, ^99^, ^100^ ^]^ Additionally, recent studies have shown that various PTMs, such as phosphorylation, acetylation, glycosylation, and ubiquitination, play critical roles in both olfactory and hearing‐related disorders (Figure 4).^[^ ^88^, ^101^, ^102^, ^103^, ^104^, ^105^, ^106^, ^107^, ^108^ ^]^

Sensory ciliopathies extend beyond defects in the interpretation of environmental cues. Primary cilia also regulate intercellular signaling pathways that, when impaired, result in ciliopathies, such as microcephaly, polycystic kidney disease, polydactyly, and obesity. Recent research has identified microtubule‐associated tyrosine carboxypeptidase (MATCAP) as a detyrosinating enzyme. In *Matcap*‐deficient mice, impaired detyrosination of α‐tubulin leads to a significant reduction in overall brain volume, recapitulating the microcephaly and associated cognitive deficits observed in humans.^[^ ^109^ ^]^ Recent studies have revealed that the palmitoylation of ciliary proteins plays a crucial role in the development of ciliopathies. Proteins such as polycystin‐1 (PC1), ARL13B, Rab3a‐interacting protein (Rab3IP), Rab8, Rab11, regulatory factor X 3 (RFX3), and progressive rod‐cone degeneration protein (PRCD) have been linked to renal cystogenesis, diabetes, and atherosclerosis through their palmitoylation.^[^ ^92^, ^110^, ^111^, ^112^, ^113^, ^114^ ^]^ Moreover, enzymes that modulate palmitoylation, such as carnitine palmitoyltransferase I (CPT1) and the depalmitoylating enzyme palmitoyl‐protein thioesterase 1 (PPT1), are involved in the development of retinopathy.^[^ ^113^, ^115^ ^]^

### 3.3 Targeting PTMs for the Treatment of Ciliopathies

Aberrant PTMs have been implicated in numerous ciliopathies, suggesting the potential of targeting PTMs for disease treatment. Several predictions regarding the disease‐causing roles of PTMs have now been validated in human disorders, often presenting phenotypes remarkably similar to those observed in mouse models. This evidence establishes a strong link between ciliopathies and aberrant PTMs, indicating that correcting imbalances in PTMs could hold promise for the therapy of ciliopathies. Indeed, recent studies have demonstrated that targeting enzymes or substrates associated with various PTMs can effectively alleviate ciliopathy‐related phenotypes. For example, various pharmacological agents targeting protein kinases have been developed for the treatment of ciliopathies. Sunitinib, a small‐molecule multitargeted receptor tyrosine kinase inhibitor approved by the FDA, has been found to effectively restrict ciliation and renal cystogenesis in autosomal dominant polycystic kidney disease (ADPKD).^[^ ^116^, ^117^ ^]^ Similarly, other multitarget protein kinase inhibitors, including sorafenib, IRAK1/4 inhibitor, and erlotinib, which are used orally for cancer treatment, have also been shown to promote cilium disassembly.^[^ ^116^ ^]^ The efficiency of these drugs in treating ciliopathies in mouse models suggests that inhibition of primary ciliogenesis may be a potential strategy for treating at least a subset of human diseases.

Furthermore, cyclin‐dependent kinase 5 (CDK5) inhibitors, such as roscovitine and its analog (S)‐CR8, have been shown to markedly suppress cystogenesis in PKD mouse models.^[^ ^118^ ^]^ This therapeutic effect is attributed to their influence on primary ciliogenesis.^[^ ^119^ ^]^ Notably, recent research has unveiled that pharmacological modulation of the fibroblast growth factor−parathyroid hormone (FGF−PTH) axis using a pan‐FGFR antagonist, AZD4547, can effectively ameliorate skeletal phenotypes associated with ciliopathies.^[^ ^120^ ^]^ Additionally, there is ongoing development of pharmacological agents targeting other PTMs for both research and therapeutic purposes in ciliopathies. For example, compounds that modulate neddylation, such as MLN4924; acetylation, such as tubacin and tubastatin A; and *O*‐GlcNAcylation, such as BZX, have been reported to influence ciliary formation and alleviate ciliopathy‐related phenotypes.^[^ ^44^, ^100^, ^121^ ^]^

While these pharmacological interventions have shown promise in preclinical models, their clinical translation faces significant hurdles. Many PTM‐modulating drugs exhibit pleiotropic effects due to the ubiquitous nature of their target enzymes. For instance, HDAC6 inhibitors like tubacin, while stabilizing ciliary structures, also regulate cytoskeletal dynamics and immune responses, potentially leading to off‐target effects. This underscores the need for more selective therapeutic strategies that can specifically target ciliary PTMs without disrupting other essential cellular processes. Additionally, while several PTM‐modifying enzymes have been identified, their substrate specificity and regulatory contexts remain incompletely characterized. For example, although phosphorylation of CEP164 and CEP83 mediated by TTBK2 is essential for ciliogenesis,^[^ ^29^ ^]^ the complete spectrum of TTBK2 substrates, particularly in pathological conditions such as Joubert syndrome, has not been fully elucidated. The regulatory complexity is further amplified by dynamic interactions between different PTMs. In motile cilia, phosphorylation of MPP9 by TTBK2 initiates a cascade of ubiquitination and degradation events required for ciliogenesis, yet the spatiotemporal regulation of this PTM crosstalk under disease conditions remains poorly understood.^[^ ^31^ ^]^ Importantly, the functional consequences of TTBK2 activity exhibit significant context‐dependent variation across different ciliopathies. In spinocerebellar ataxia type 11, truncated TTBK2 variants act as dominant‐negative mutants that impair ciliary stability and disrupt Sonic Hedgehog signaling.^[^ ^27^ ^]^ Conversely, in medulloblastoma, TTBK2 maintains cilia‐dependent proliferative signaling through mechanisms that evade HUWE1‐mediated degradation.^[^ ^122^ ^]^ Additional complexity arises from multi‐layered PTM networks, as exemplified by the macrophage migration inhibitory factor (MIF)‐phosphatidylinositol‐5‐phosphate 4‐kinase type 2 alpha (PIP4K2α)‐TTBK2 axis. Here, phosphorylation of MIF at serine 91 by PIP4K2a coordinately regulates transcriptional programs and TTBK2 recruitment to basal bodies.^[^ ^123^ ^]^ These findings collectively demonstrate that non‐selective kinase inhibition strategies are unlikely to succeed, given the diverse roles of TTBK2 in ciliary initiation, maintenance, and signaling across different disease contexts. Consequently, future therapeutic development must consider not only substrate diversity but also the precise spatiotemporal regulation of PTM networks specific to each pathological condition, thereby minimizing potential off‐target effects.

To overcome these limitations, future therapeutic development must address two key challenges: achieving sufficient target specificity to minimize off‐target effects and developing delivery systems that can precisely target affected tissues. This is particularly crucial given that many PTM‐modifying enzymes participate in multiple cellular pathways beyond ciliary function. Given these challenges, the development of small‐molecule drugs targeting distinct substrate proteins in different disease states becomes particularly crucial. However, most of these substrate proteins are structural components that typically lack well‐defined binding pockets for conventional small‐molecule interventions. Consequently, the discovery and development of potent small molecules that precisely target PTM‐related substrate proteins represent challenging issues in the treatment of ciliopathies. In recent years, several promising therapeutic strategies have emerged, notably the targeted protein degradation (TPD) technology. The TPD technology holds the potential to effectively reduce pathogenic proteins that have been difficult to address with traditional small molecules, and demonstrates considerable promise in treating various diseases, such as cancer and ocular disorders.^[^ ^124^ ^]^ By leveraging this technology, it becomes feasible to target “undruggable” proteins related to PTMs that have been implicated in ciliopathies, thus providing new avenues for therapeutic intervention.

## 4 Conclusion and Perspective

The intricate roles of PTMs in ciliary structure and function underscore the complexity of ciliary biology and have important implications in human diseases. As delineated in this review, PTMs such as palmitoylation, glutamylation, UFMylation, and glycylation are pivotal in regulating ciliary formation and homeostasis. These modifications orchestrate a multitude of cellular processes, bridging ciliary structure and signaling with broader cellular and organismal physiology. In particular, emerging PTMs like UFMylation and *O*‐GlcNAcylation have recently been implicated in ciliary function, though their regulatory mechanisms and disease relevance remain underexplored. The UFMylation cascade, mediated by the E1 activating enzyme UBA5, E2 conjugating enzyme UFC1, and E3 ligase UFL1,^[^ ^125^ ^]^ has been shown to modify key ciliary proteins such as KIF11, which is essential for photoreceptor cilium maintenance.^[^ ^81^, ^82^ ^]^ However, fundamental questions persist, such as how is the UFMylation machinery spatially organized at the basal body or transition zone? Do motile cilia employ distinct UFMylation substrates compared to primary cilia? Is UFMylation dysregulated in syndromic ciliopathies like Bardet‐Biedl syndrome, where ubiquitin‐proteasome system defects are already implicated? Parallel questions apply to *O*‐GlcNAcylation, a nutrient‐sensitive PTM that modulates ciliary length but lacks comprehensive substrate maps in ciliary compartments.^[^ ^100^, ^126^ ^]^

Our expanding knowledge of these modifications highlights their critical contributions to the pathogenesis of ciliopathies, providing novel insights into the molecular underpinnings of these disorders. The known connections between PTMs and ciliopathies will continue to increase, potentially encompassing diseases not traditionally classified as ciliopathies, such as cancer and congenital heart disorders. The discovery of rare disease variants in essential genes has the potential to reveal unexpected roles in ciliogenesis, thereby elucidating their physiological and pathological functions in tissue homeostasis and ciliary diseases. For example, mutations in KIF11, a crucial regulator of mitotic spindle formation and maintenance, have been associated with cancer, microcephaly, familial exudative vitreoretinopathy, and ciliogenesis disorders.^[^ ^127^, ^128^, ^129^, ^130^ ^]^ These findings prompted investigations into the role of UFMylation of KIF11 in maintaining the integrity of photoreceptor cilia and retinal homeostasis, thereby enhancing our understanding of the intricate regulatory networks involved in ciliary function and associated diseases.^[^ ^82^ ^]^ Intriguingly, *UBA5* mutations cause developmental encephalopathy with microcephaly,^[^ ^131^ ^]^ a phenotype overlapping with ciliopathies, suggesting potential ciliary roles for UFMylation beyond KIF11 modulation.

Despite advances in identifying the functions of various PTMs in ciliary regulation, much remains unknown about alterations in PTM‐related enzymes and substrate proteins involved in tissue homeostasis and ciliopathies. For example, although acetylation and detyrosination influence the stability and function of cilia, the specific enzymes involved and their regulatory mechanisms in ciliopathies are still being elucidated. For example, while detyrosinated tubulin is known to be enriched on B‐tubules of ciliary axonemes, the functional specialization of tubulin tyrosine ligase (TTL) in ciliogenesis remains enigmatic. This is particularly intriguing given that TTL‐knockout mice exhibit perinatal lethality with severe neuronal organization defects,^[^ ^132^ ^]^ yet the potential roles of this enzyme in ciliary assembly, such as regulating the tyrosination/detyrosination balance at basal bodies or modulating IFT particle recruitment, remain completely unexplored. The recent identification of tubulin detyrosinase VashL as a key regulator for ciliary growth by sorting of IFT trains on axonemal microtubule doublets, highlighting these PTMs in ciliary biogenesis.^[^ ^133^ ^]^ Given these challenges, the identification of novel ciliary proteins and their modification states is of paramount importance. For example, exploring the role of cilium‐specific kinases and phosphatases could uncover new regulatory layers of ciliary function. Similarly, exploring how lipid modifications, such as farnesylation, influence the membrane association of ciliary components could offer deeper insights into ciliary membrane dynamics and integrity. Perhaps most importantly, understanding how the crosstalk between different PTMs coordinates ciliogenesis and contributes to the pathophysiology of ciliopathies represents a key scientific question that deserves thorough and in‐depth investigation.

To better understand the functional consequences of PTM crosstalk in cilia, mass spectrometry (MS)‐based proteomics has emerged as a promising tool for the discovery and detailed characterization of PTMs.^[^ ^134^ ^]^ MS‐based proteomics encompasses two primary approaches: bottom‐up and top‐down proteomics. The bottom‐up approach, which involves protein digestion into peptides followed by liquid chromatography and tandem MS analysis, has been widely used due to its high sensitivity and compatibility with complex samples. However, the top‐down approach, which avoids protein digestion and directly analyzes intact proteins, provides a more comprehensive view of proteoforms, distinct protein variants carrying specific combinations of PTMs.^[^ ^135^ ^]^ This capability is particularly valuable in studying PTM crosstalk, a critical factor in the pathogenesis of ciliopathies. For instance, top‐down proteomics has been successfully applied to analyze intact forms of histones, revealing complex PTM patterns that regulate chromatin dynamics and gene expression.^[^ ^136^, ^137^ ^]^ Additionally, this approach has been instrumental in identifying specific proteoforms of bacterial proteins, such as the PilE in *Neisseria meningitidis*, which is tightly associated with the ability of the pathogen to cross epithelial barriers and evade immune detection.^[^ ^138^ ^]^ Recent advancements in MS technology, such as improved resolution and sensitivity, have further enhanced the utility of top‐down proteomics in clinical and research settings,^[^ ^139^, ^140^, ^141^ ^]^ paving the way for a deeper understanding of ciliopathies and the development of targeted therapeutic strategies.

Enzymes that catalyze PTMs are attractive targets for drug development.^[^ ^142^ ^]^ Small‐molecule inhibitors or biological agents targeting these enzymes show promise for treating pathologies linked to aberrant PTMs. However, developing selective agents remains challenging, owing to the complexity of protein structures. Precise 3D structures are critical for understanding protein function and optimizing drug binding, but obtaining these structures has been a major obstacle.^[^ ^143^ ^]^ Recent advances in structural biology and artificial intelligence (AI) offer potential solutions to overcome these challenges.^[^ ^144^ ^]^ AI algorithms, such as AlphaFold3, can predict protein structures, including protein−ligand and antibody−protein interactions, with near−near‐experimental accuracy and a 50% improvement over traditional methods in the PoseBusters benchmark.^[^ ^145^ ^]^ This breakthrough makes AlphaFold3 the first AI system to surpass physics‐based tools, enabling faster and more accurate drug target identification. This computational tool, combined with techniques such as cryo‐electron microscopy and X‐ray crystallography, facilitates the design of potent and selective agents that target enzymes catalyzing PTMs. Additionally, unlike traditional protein‐based drugs, gene therapy enables the long‐term expression of therapeutic proteins within cells or tissues.^[^ ^146^ ^]^ This can provide sustained benefits over time. Among gene delivery vectors, adeno‐associated virus (AAV) vectors are particularly favored due to their low immunogenicity, low cytotoxicity, and minimal risk of genomic integration. Several AAV‐based gene therapies have already received clinical approval.^[^ ^147^ ^]^ Further studies are warranted to explore the potential of AAV‐based gene therapies in addressing ciliopathies linked to aberrant PTMs.

## Conflict of Interest

The authors declare no conflict of interest.

## Author Contributions

J.R. wrote the manuscript and drew the figures. J.Z. supervised the project and edited the manuscript.

## References

[1] K. I. Hilgendorf , B. R. Myers , J. F. Reiter , Nat. Rev. Mol. Cell Biol. 2024, 25, 555. 38366037 10.1038/s41580-023-00698-5 PMC11199107

[2] P. Mill , S. T. Christensen , L. B. Pedersen , Nat. Rev. Genet. 2023, 24, 421. 37072495 10.1038/s41576-023-00587-9 PMC7615029

[3] J. Wallmeier , K. G. Nielsen , C. E. Kuehni , J. S. Lucas , M. W. Leigh , M. A. Zariwala , H. Omran , M. Ciliopathies , Nat. Rev. Dis. Primers 2020, 6, 77. 32943623 10.1038/s41572-020-0209-6

[4] A. L. Moran , L. Louzao‐Martinez , D. P. Norris , D. J. M. Peters , O. E. Blacque , Nat. Rev. Nephrol. 2024, 20, 83. 37872350 10.1038/s41581-023-00773-2

[5] S. K. Suciu , T. Caspary , Semin. Cell Dev. Biol. 2021, 110, 34. 32732132 10.1016/j.semcdb.2020.07.014 PMC7855023

[6] D. Wloga , E. Joachimiak , P. Louka , J. Gaertig , Cold Spring Harb. Perspect. Biol. 2017, 9, 028159. 10.1101/cshperspect.a028159 PMC5453388 28003186

[7] K. Roy , S. Jerman , L. Jozsef , T. McNamara , G. Onyekaba , Z. Sun , E. P. Marin , J. Biol. Chem. 2017, 292, 17703. 28848045 10.1074/jbc.M117.792937 PMC5663873

[8] T. Chaya , Y. Omori , R. Kuwahara , T. Furukawa , EMBO J. 2014, 33, 1227. 24797473 10.1002/embj.201488175 PMC4198026

[9] M. M. Magiera , P. Singh , S. Gadadhar , C. Janke , Cell 2018, 173, 1323. 29856952 10.1016/j.cell.2018.05.018

[10] S. Gadadhar , G. Alvarez Viar , J. N. Hansen , A. Gong , A. Kostarev , C. Ialy‐Radio , S. Leboucher , M. Whitfield , A. Ziyyat , A. Touré , L. Alvarez , G. Pigino , C. Janke , Science 2021, 371, 4914. 10.1126/science.abd4914 PMC7612590 33414192

[11] J. Ran , M. Liu , J. Feng , H. Li , H. Ma , T. Song , Y. Cao , P. Zhou , Y. Wu , Y. Yang , Y. Yang , F. Yu , H. Guo , L. Zhang , S. Xie , D. Li , J. Gao , X. Zhang , X. Zhu , J. Zhou , Dev. Cell 2020, 53, 287. 32275885 10.1016/j.devcel.2020.03.010

[12] K. Kasahara , M. Inagaki , Trends Cell Biol. 2021, 31, 954. 34420822 10.1016/j.tcb.2021.07.009

[13] D. Hossain , W. Y. Tsang , Semin. Cell Dev. Biol. 2019, 93, 145. 30213760 10.1016/j.semcdb.2018.09.005

[14] H. Ishikawa , J. Thompson , J. R. Yates 3rd , W. F. Marshall , Curr. Biol. 2012, 22, 414. 22326026 10.1016/j.cub.2012.01.031 PMC3298568

[15] Q. Liu , G. Tan , N. Levenkova , T. Li , E. N. Pugh , J. J. Rux , D. W. Speicher , E. A. Pierce , Mol. Cell. Proteomics 2007, 6, 1299. 17494944 10.1074/mcp.M700054-MCP200 PMC2128741

[16] B. H. Villumsen , J. R. Danielsen , L. Povlsen , K. B. Sylvestersen , A. Merdes , P. Beli , Y.‐G. Yang , C. Choudhary , M. L. Nielsen , N. Mailand , S. Bekker‐Jensen , EMBO J. 2013, 32, 3029. 24121310 10.1038/emboj.2013.223 PMC3844950

[17] T. Douanne , G. André‐Grégoire , A. Thys , K. Trillet , J. Gavard , N. Bidère , Cell Rep. 2019, 27, 1657. 31067453 10.1016/j.celrep.2019.04.036

[18] K. J. Han , Z. Wu , C. G. Pearson , J. Peng , K. Song , C. W. Liu , J. Cell Sci. 2019, 132, 145. 10.1242/jcs.221663 PMC6362394 30584065

[19] T. Eguether , M. A. Ermolaeva , Y. Zhao , M. C. Bonnet , A. Jain , M. Pasparakis , G. Courtois , A.‐M. Tassin , Nat. Commun. 2014, 5, 4585. 25134987 10.1038/ncomms5585

[20] X. Li , N. Song , L. Liu , X. Liu , X. Ding , X. Song , S. Yang , L. Shan , X. Zhou , D. Su , Y. Wang , Q. Zhang , C. Cao , S. Ma , N. Yu , F. Yang , Y. Wang , Z. Yao , Y. Shang , L. Shi , Nat. Commun. 2017, 8, 14866. 28361952 10.1038/ncomms14866 PMC5380967

[21] P. Wang , J. Xia , L. Zhang , S. Zhao , S. Li , H. Wang , S. Cheng , H. Li , W. Yin , D. Pei , X. Shu , Cells 2019, 8, 1335. 31671755 10.3390/cells8111335 PMC6912348

[22] I.‐H. Lin , Y.‐R. Li , C.‐H. Chang , Y.‐W. Cheng , Y.‐T. Wang , Y.‐S. Tsai , P.‐Y. Lin , C.‐H. Kao , T.‐Y. Su , C.‐S. Hsu , C.‐Y. Tung , P.‐H. Hsu , O. Ayrault , B.‐C. Chung , J.‐W. Tsai , W.‐J. Wang , Cell Death 2024, 31, 1349. 10.1038/s41418-024-01325-2 PMC11445238 38879724

[23] J. Li , V. D'Angiolella , E. S. Seeley , S. Kim , T. Kobayashi , W. Fu , E. I. Campos , M. Pagano , B. D. Dynlacht , Nature 2013, 495, 255. 23486064 10.1038/nature11941 PMC3815529

[24] D. Hossain , Y. Javadi Esfehani , A. Das , W. Y. Tsang , EMBO Rep. 2017, 18, 632. 28242748 10.15252/embr.201642377 PMC5376967

[25] V. D'Angiolella , V. Donato , S. Vijayakumar , A. Saraf , L. Florens , M. P. Washburn , B. Dynlacht , M. Pagano , Nature 2010, 466, 632. 20596027 10.1038/nature09140 PMC2946399

[26] A. Hori , K. Barnouin , A. P. Snijders , T. Toda , EMBO Rep. 2016, 17, 326. 26755742 10.15252/embr.201541432 PMC4772974

[27] S. C. Goetz , K. F. Liem, Jr. , K. V. Anderson , Cell 2012, 151, 847. 23141541 10.1016/j.cell.2012.10.010 PMC3496184

[28] L. Čajánek , E. A. Nigg , Proc. Natl. Acad. Sci. U.S.A. 2014, 111, 326. 24982133 10.1073/pnas.1401777111 PMC4104846

[29] C.‐H. Lo , I.‐H. Lin , T. T. Yang , Y.‐C. Huang , B. E. Tanos , P.‐C. Chou , C.‐W. Chang , Y.‐G. Tsay , J.‐C. Liao , W.‐J. Wang , J. Cell Biol. 2019, 218, 3489. 31455668 10.1083/jcb.201811142 PMC6781440

[30] S. Kuhns , K. N. Schmidt , J. Reymann , D. F. Gilbert , A. Neuner , B. Hub , R. Carvalho , P. Wiedemann , H. Zentgraf , H. Erfle , U. Klingmüller , M. Boutros , G. Pereira , J. Cell Biol. 2013, 200, 505. 23400999 10.1083/jcb.201206013 PMC3575539

[31] N. Huang , D. Zhang , F. Li , P. Chai , S. Wang , J. Teng , J. Chen , Nat. Commun. 2018, 9, 4511. 30375385 10.1038/s41467-018-06990-9 PMC6207757

[32] Y. Bobinnec , A. Khodjakov , L. M. Mir , C. L. Rieder , B. Eddé , M. Bornens , J. Cell Biol. 1998, 143, 1575. 9852152 10.1083/jcb.143.6.1575 PMC2132987

[33] L. Wang , S. C. Paudyal , Y. Kang , M. Owa , F.‐X. Liang , A. Spektor , H. Knaut , I. Sánchez , B. D. Dynlacht , Cell Res. 2022, 32, 190. 34782749 10.1038/s41422-021-00584-9 PMC8807603

[34] A. D. Junker , A. W. J. Soh , E. T. O'Toole , J. B. Meehl , M. Guha , M. Winey , J. E. Honts , J. Gaertig , C. G. Pearson , J. Cell Sci. 2019, 13, 2233726. 10.1242/jcs.233726 PMC6703707 31243050

[35] S. W. L'Hernault , J. L. Rosenbaum , J. Cell Biol. 1983, 97, 258. 6863393 10.1083/jcb.97.1.258 PMC2112491

[36] E. Nogales , M. Whittaker , R. A. Milligan , K. H. Downing , Cell 1999, 96, 79. 9989499 10.1016/s0092-8674(00)80961-7

[37] J. S. Akella , D. Wloga , J. Kim , N. G. Starostina , S. Lyons‐Abbott , N. S. Morrissette , S. T. Dougan , E. T. Kipreos , J. Gaertig , Nature 2010, 467, 218. 20829795 10.1038/nature09324 PMC2938957

[38] N. Kalebic , S. Sorrentino , E. Perlas , G. Bolasco , C. Martinez , P. A. Heppenstall , Nat. Commun. 2013, 4, 1962. 23748901 10.1038/ncomms2962

[39] T. Shida , J. G. Cueva , Z. Xu , M. B. Goodman , M. V. Nachury , Proc. Natl. Acad. Sci. U.S.A. 2010, 107, 21517. 21068373 10.1073/pnas.1013728107 PMC3003046

[40] X. Zhou , L. X. Fan , K. Li , R. Ramchandran , J. P. Calvet , X. Li , Hum. Mol. Genet. 2014, 23, 1644. 24203696 10.1093/hmg/ddt556 PMC3929098

[41] J. Ran , Y. Yang , D. Li , M. Liu , J. Zhou , Sci. Rep. 2015, 5, 12917. 26246421 10.1038/srep12917 PMC4526867

[42] F. Yu , J. Ran , J. Zhou , Trends Pharmacol. Sci. 2016, 37, 114. 26651415 10.1016/j.tips.2015.11.002

[43] S. A. Gradilone , B. N. Radtke , P. S. Bogert , B. Q. Huang , G. B. Gajdos , N. F. LaRusso , Cancer Res. 2013, 73, 2259. 23370327 10.1158/0008-5472.CAN-12-2938 PMC3768151

[44] J. Ran , Y. Zhang , S. Zhang , H. Li , L. Zhang , Q. Li , J. Qin , D. Li , L. Sun , S. Xie , X. Zhang , L. Liu , M. Liu , J. Zhou , Adv. Sci. 2022, 9, 2105365. 10.1002/advs.202105365 PMC9313505 35619548

[45] A. Sánchez de Diego , A. A. Guerrero , A. C. Martínez , K. H. van Wely , Nat. Commun. 2014, 5, 3500. 24667272 10.1038/ncomms4500 PMC3973121

[46] J. Ran , J. Zhou , Acta. Pharmacol. Sin. 2020, 41, 1410. 32753732 10.1038/s41401-020-0486-3 PMC7656575

[47] M. B. Grau , G. Gonzalez Curto , C. Rocha , M. M. Magiera , P. M. Sousa , T. Giordano , N. Spassky , C. Janke , J. Cell Biol. 2013, 202, 441. 23897886 10.1083/jcb.201305041 PMC3734080

[48] K. Ikegami , S. Sato , K. Nakamura , L. E. Ostrowski , M. Setou , Proc. Natl. Acad. Sci. U.S.A. 2010, 107, 10490. 20498047 10.1073/pnas.1002128107 PMC2890849

[49] C. Gagnon , D. White , J. Cosson , P. Huitorel , B. Eddé , E. Desbruyères , L. Paturle‐Lafanechère , L. Multigner , D. Job , C. Cibert , J. Cell Sci. 1996, 109, 1545. 8799841 10.1242/jcs.109.6.1545

[50] T. Giordano , S. Gadadhar , S. Bodakuntla , J. Straub , S. Leboucher , G. Martinez , W. Chemlali , C. Bosc , A. Andrieux , I. Bieche , C. Arnoult , S. Geimer , C. Janke , J. Cell Sci. 2019, 132, 226951. 10.1242/jcs.226951 30635446

[51] T. Kubo , H. A. Yanagisawa , T. Yagi , M. Hirono , R. Kamiya , Curr. Biol. 2010, 20, 441. 20188560 10.1016/j.cub.2009.12.058

[52] N. Pathak , C. A. Austin , I. A. Drummond , J. Biol. Chem. 2011, 286, 11685. 21262966 10.1074/jbc.M110.209817 PMC3064220

[53] S. Gadadhar , H. Dadi , S. Bodakuntla , A. Schnitzler , I. Bièche , F. Rusconi , C. Janke , J. Cell Biol. 2017, 216, 2701. 28687664 10.1083/jcb.201612050 PMC5584158

[54] R. O'Hagan , M. Silva , K. C. Q. Nguyen , W. Zhang , S. Bellotti , Y. H. Ramadan , D. H. Hall , M. M. Barr , Curr. Biol. 2017, 27, 3430. 29129530 10.1016/j.cub.2017.09.066 PMC5698134

[55] M. B. Grau , C. Masson , S. Gadadhar , C. Rocha , O. Tort , P. M. Sousa , S. Vacher , I. Bieche , C. Janke , J. Cell Sci. 2017, 130, 938. 28104815 10.1242/jcs.199091

[56] J. E. Lee , J. L. Silhavy , M. S. Zaki , J. Schroth , S. L. Bielas , S. E. Marsh , J. Olvera , F. Brancati , M. Iannicelli , K. Ikegami , A. M. Schlossman , B. Merriman , T. Attié‐Bitach , C. V. Logan , I. A. Glass , A. Cluckey , C. M. Louie , J. H. Lee , H. R. Raynes , I. Rapin , I. P. Castroviejo , M. Setou , C. Barbot , E. Boltshauser , S. F. Nelson , F. Hildebrandt , C. A. Johnson , D. A. Doherty , E. M. Valente , J. G. Gleeson , Nat. Genet. 2012, 44, 193. 22246503 10.1038/ng.1078 PMC3267856

[57] S. M. Ki , J. H. Kim , S. Y. Won , S. J. Oh , I. Y. Lee , Y.‐K. Bae , K. W. Chung , B.‐O. Choi , B. Park , E.‐J. Choi , J. E. Lee , EMBO Rep. 2020, 21, 48290. 10.15252/embr.201948290 PMC7001496 31885126

[58] L. Paturle‐Lafanechère , M. Manier , N. Trigault , F. Pirollet , H. Mazarguil , D. Job , J. Cell Sci. 1994, 107, 1529. 7962195 10.1242/jcs.107.6.1529

[59] M. Sirajuddin , L. M. Rice , R. D. Vale , Nat. Cell Biol. 2014, 16, 335. 24633327 10.1038/ncb2920 PMC4117587

[60] H. van den Hoek , N. Klena , M. A. Jordan , G. Alvarez Viar , R. D. Righetto , M. Schaffer , P. S. Erdmann , W. Wan , S. Geimer , J. M. Plitzko , W. Baumeister , G. Pigino , V. Hamel , P. Guichard , B. D. Engel , Science 2022, 377, 543. 35901159 10.1126/science.abm6704

[61] L. Li , J. Ran , Cell Death Dis. 2024, 15, 47. 38218748 10.1038/s41419-024-06428-9 PMC10787775

[62] S. E. Lacey , G. Pigino , Nat. Rev. Mol. Cell Biol. 2025, 26, 175. 39537792 10.1038/s41580-024-00797-x

[63] M. Peralta , L. Ortiz Lopez , K. Jerabkova , T. Lucchesi , B. Vitre , D. Han , L. Guillemot , C. Dingare , I. Sumara , N. Mercader , V. Lecaudey , B. Delaval , S. M. Meilhac , J. Vermot , Cell Rep. 2020, 32, 107932. 32698004 10.1016/j.celrep.2020.107932

[64] M. S. Lee , K. S. Hwang , H. W. Oh , K. Ji‐Ae , H. T. Kim , H. S. Cho , J. J. Lee , J. Yeong Ko , J. H. Choi , Y. M. Jeong , K. H. You , J. Kim , D. S. Park , K. H. Nam , S. Aizawa , H. Kiyonari , G. Shioi , J. H. Park , W. Zhou , N. S. Kim , C. H. Kim , Dev. Biol. 2015, 400, 248. 25722189 10.1016/j.ydbio.2015.02.009 PMC4385464

[65] Y. Omori , T. Chaya , K. Katoh , N. Kajimura , S. Sato , K. Muraoka , S. Ueno , T. Koyasu , M. Kondo , T. Furukawa , Proc. Natl. Acad. Sci. U.S.A. 2010, 107, 22671. 21148103 10.1073/pnas.1009437108 PMC3012466

[66] R. K. Özgül , A. M. Siemiatkowska , D. Yücel , C. A. Myers , R. W. J. Collin , M. N. Zonneveld , A. Beryozkin , E. Banin , C. B. Hoyng , L. I. van den Born , R. Bose , W. Shen , D. Sharon , F. P. M. Cremers , B. J. Klevering , A. I. den Hollander , J. C. Corbo , Am. J. Hum. Genet. 2011, 89, 253. 21835304 10.1016/j.ajhg.2011.07.005 PMC3155188

[67] B. A. Tucker , T. E. Scheetz , R. F. Mullins , A. P. DeLuca , J. M. Hoffmann , R. M. Johnston , S. G. Jacobson , V. C. Sheffield , E. M. Stone , Proc. Natl. Acad. Sci. U.S.A. 2011, 108. 10.1073/pnas.1108918108 PMC3161526 21825139

[68] K. Nakamura , T. Noguchi , M. Takahara , Y. Omori , T. Furukawa , Y. Katoh , K. Nakayama , J. Biol. Chem. 2020, 295, 13363. 32732286 10.1074/jbc.RA120.014142 PMC7504932

[69] Z. Fu , C. D. Gailey , E. J. Wang , D. L. Brautigan , FEBS Lett. 2019, 593, 2990. 31506943 10.1002/1873-3468.13600 PMC6848764

[70] P. Lahiry , J. Wang , J. F. Robinson , J. P. Turowec , D. W. Litchfield , M. B. Lanktree , G. B. Gloor , E. G. Puffenberger , K. A. Strauss , M. B. Martens , D. A. Ramsay , C. A. Rupar , V. Siu , R. A. Hegele , Am. J. Hum. Genet. 2009, 84, 134. 19185282 10.1016/j.ajhg.2008.12.017 PMC2668000

[71] M. M. Oud , C. Bonnard , D. A. Mans , U. Altunoglu , S. Tohari , A. Y. J. Ng , A. Eskin , H. Lee , C. A. Rupar , N. P. de Wagenaar , K. M. Wu , P. Lahiry , G. J. Pazour , S. F. Nelson , R. A. Hegele , R. Roepman , H. Kayserili , B. Venkatesh , V. M. Siu , B. Reversade , H. H. Arts , Cilia 2016, 5, 8. 27069622 10.1186/s13630-016-0029-1 PMC4827216

[72] S. Paige Taylor , M. Kunova Bosakova , M. Varecha , L. Balek , T. Barta , L. Trantirek , I. Jelinkova , I. Duran , I. Vesela , K. N. Forlenza , J. H. Martin , A. Hampl , M. Bamshad , D. Nickerson , M. L. Jaworski , J. Song , H. W. Ko , D. H. Cohn , D. Krakow , P. Krejci , Hum. Mol. Genet. 2016, 25, 3998. 27466187 10.1093/hmg/ddw240 PMC5291234

[73] R. Hong , Y. Tan , X. Tian , Z. Huang , J. Wang , H. Ni , J. Yang , W. Bu , S. Yang , T. Li , F. Yu , W. Zhong , T. Sun , X. Wang , D. Li , M. Liu , Y. Yang , J. Zhou , EMBO Rep. 2024, 25, 1055. 38351372 10.1038/s44319-024-00092-y PMC10933415

[74] O. Mercey , S. Gadadhar , M. M. Magiera , L. Lebrun , C. Kostic , A. Moulin , Y. Arsenijevic , C. Janke , P. Guichard , V. Hamel , EMBO J. 2024, 43, 6679. 39528655 10.1038/s44318-024-00284-1 PMC11649768

[75] O. Mercey , S. Mukherjee , P. Guichard , V. Hamel , Curr. Opin. Cell Biol. 2024, 88, 102361. 38648677 10.1016/j.ceb.2024.102361

[76] K. Park , M. R. Leroux , EMBO Rep. 2022, 23, 55420. 10.15252/embr.202255420 PMC9724682 36408840

[77] X. Wu , M. Xu , M. Geng , S. Chen , P. J. Little , S. Xu , J. Weng , Signal Transduct. Target. Ther. 2023, 8, 438. 37244925 10.1038/s41392-023-01439-y PMC10224996

[78] K. N. Rao , W. Zhang , L. Li , M. Anand , H. Khanna , Hum. Mol. Genet. 2016, 25, 2005. 28172980 10.1093/hmg/ddw281 PMC6078598

[79] D.‐H. Hong , B. Pawlyk , M. Sokolov , K. J. Strissel , J. Yang , B. Tulloch , A. F. Wright , V. Y. Arshavsky , T. Li , Invest Ophthalmol. Vis. Sci. 2003, 44, 2413. 12766038 10.1167/iovs.02-1206

[80] X. Sun , J. H. Park , J. Gumerson , Z. Wu , A. Swaroop , H. Qian , A. Roll‐Mecak , T. Li , Proc. Natl. Acad. Sci. U.S.A. 2016, 113, 2925. 10.1073/pnas.1523201113 PMC4889371 27162334

[81] X. Zhou , S. J. Mahdizadeh , M. L. Gallo , L. A. Eriksson , E. Chevet , E. Lafont , Trends Biochem. Sci. 2024, 49. 10.1016/j.tibs.2023.10.004 37945409

[82] J. Ran , G. Guo , S. Zhang , Y. Zhang , L. Zhang , D. Li , S. Wu , Y. Cong , X. Wang , S. Xie , H. Zhao , H. Liu , G. Ou , X. Zhu , J. Zhou , M. Liu , Adv. Sci. 2024, 11, 2400569. 10.1002/advs.202400569 PMC11220646 38666385

[83] S. Apionishev , N. M. Katanayeva , S. A. Marks , D. Kalderon , A. Tomlinson , Nat. Cell Biol. 2005, 7, 86. 15592457 10.1038/ncb1210

[84] J. Jia , C. Tong , B. Wang , L. Luo , J. Jiang , Nature 2004, 432, 1045. 15616566 10.1038/nature03179

[85] Y. Chen , S. Li , C. Tong , Y. Zhao , B. Wang , Y. Liu , J. Jia , J. Jiang , Genes Dev. 2010, 24, 2054. 20844016 10.1101/gad.1948710 PMC2939367

[86] Y. Zhao , C. Tong , J. Jiang , Nature 2007, 450, 252. 17960137 10.1038/nature06225

[87] G. Ma , S. Li , Y. Han , S. Li , T. Yue , B. Wang , J. Jiang , Dev. Cell 2016, 39, 438. 27746045 10.1016/j.devcel.2016.09.014 PMC5121078

[88] J. C. McIntyre , A. M. Joiner , L. Zhang , J. Iñiguez‐Lluhí , J. R. Martens , J. Cell Sci. 2015, 128, 1934. 25908845 10.1242/jcs.164673 PMC4457158

[89] V. Cantagrel , J. L. Silhavy , S. L. Bielas , D. Swistun , S. E. Marsh , J. Y. Bertrand , S. Audollent , T. Attié‐Bitach , K. R. Holden , W. B. Dobyns , D. Traver , L. Al‐Gazali , B. R. Ali , T. H. Lindner , T. Caspary , E. A. Otto , F. Hildebrandt , I. A. Glass , C. V. Logan , C. A. Johnson , C. Bennett , F. Brancati , International Joubert Syndrome Related Disorders Study Group , E. M. Valente , C. G. Woods , J. G. Gleeson , Am. J. Hum. Genet. 2008, 83. 10.1016/j.ajhg.2008.06.023 PMC2495072 18674751

[90] T. Caspary , C. E. Larkins , K. V. Anderson , Dev. Cell 2007, 12, 767. 17488627 10.1016/j.devcel.2007.03.004

[91] S. Cevik , Y. Hori , O. I. Kaplan , K. Kida , T. Toivenon , C. Foley‐Fisher , D. Cottell , T. Katada , K. Kontani , O. E. Blacque , J. Cell Biol. 2010, 188, 953. 20231383 10.1083/jcb.200908133 PMC2845074

[92] Y. Tan , Z. Huang , Y. Jin , J. Wang , H. Fan , Y. Liu , L. Zhang , Y. Wu , P. Liu , T. Li , J. Ran , H. Tian , S. M. Lam , M. Liu , J. Zhou , Y. Yang , Nat. Commun. 2024, 15, 8273. 39333556 10.1038/s41467-024-52621-x PMC11437155

[93] N. Pathak , C. A. Austin‐Tse , Y. Liu , A. Vasilyev , I. A. Drummond , Mol. Biol. Cell 2014, 25. 10.1091/mbc.E13-01-0033 PMC4055263 24743595

[94] G. A. Viar , N. Klena , F. Martino , A. P. Nievergelt , D. Bolognini , P. Capasso , G. Pigino , Curr. Biol. 2024, 34, 4464. 39270640 10.1016/j.cub.2024.08.021 PMC11466076

[95] H. Zhang , C. Hanke‐Gogokhia , L. Jiang , X. Li , P. Wang , C. D. Gerstner , J. M. Frederick , Z. Yang , W. Baehr , FASEB J. 2015, 29, 932. 25422369 10.1096/fj.14-257915 PMC4422365

[96] U. Bruning , F. Morales‐Rodriguez , J. Kalucka , J. Goveia , F. Taverna , K. C. S. Queiroz , C. Dubois , A. R. Cantelmo , R. Chen , S. Loroch , E. Timmerman , V. Caixeta , K. Bloch , L.‐C. Conradi , L. Treps , A. Staes , K. Gevaert , A. Tee , M. Dewerchin , C. F. Semenkovich , F. Impens , B. Schilling , E. Verdin , J. V. Swinnen , J. L. Meier , R. A. Kulkarni , A. Sickmann , B. Ghesquière , L. Schoonjans , X. Li , et al., Cell Metab. 2018, 28, 866. 30146486 10.1016/j.cmet.2018.07.019 PMC8057116

[97] Z. Niu , C. Chen , S. Wang , C. Lu , Z. Wu , A. Wang , J. Mo , J. Zhang , Y. Han , Y. Yuan , Y. Zhang , Y. Zang , C. He , X. Bai , S. Tian , G. Zhai , X. Wu , K. Zhang , Nat. Commun. 2024, 15, 3561. 38670996 10.1038/s41467-024-47900-6 PMC11053077

[98] S. Yang , H. Liu , H. Ni , L. Jiang , M. Yang , Q. Chen , J. Zhou , F. Yu , J. Genet Genomics 2023, 50, 486. 36796536 10.1016/j.jgg.2023.02.003

[99] S. Yang , F. Yu , M. Yang , H. Ni , W. Bu , H. Yin , J. Yang , W. Wang , D. Zhai , X. Wu , N. Ma , T. Li , H. Hao , J. Ran , T. Song , D. Li , S. Yoshida , Q. Lu , Y. Yang , J. Zhou , M. Liu , Adv. Sci. 2024, 11, 2404067. 10.1002/advs.202404067 PMC11615780 39373352

[100] F. Yu , S. Guo , T. Li , J. Ran , W. Zhao , D. Li , M. Liu , X. Yan , X. Yang , X. Zhu , J. Zhou , Cell Res. 2019, 29, 171. 30429527 10.1038/s41422-018-0114-7 PMC6355847

[101] H. Yu , D. Liu , Y. Zhang , R. Tang , X. Fan , S. Mao , L. Lv , F. Chen , H. Qin , Z. Zhang , D. MF van Aalten , B. Yang , K. Yuan , Elife 2024, 13. 10.7554/eLife.91269 PMC11018347 38619103

[102] J. G. Dishart , C. L. Pender , K. Shen , H. Zhang , M. Ly , M. B. Webb , A. Dillin , Sci. Adv. 2024, 10. 10.1126/sciadv.adn0014 PMC11192085 38905346

[103] R. Singh , H. Meng , T. Shen , L. E. V. Lumahan , S. Nguyen , H. Shen , S. Dasgupta , L. Qin , D. Karri , B. Zhu , F. Yang , C. Coarfa , B. W. O'Malley , P. Yi , Proc. Natl. Acad. Sci. U.S.A. 2023, 120, 2218229120. 10.1073/pnas.2218229120 PMC10193960 37155905

[104] L. Bieniussa , I. Jain , M. Bosch Grau , L. Juergens , R. Hagen , C. Janke , K. Rak , Semin. Cell Dev. Biol. 2023, 137, 74. 35144861 10.1016/j.semcdb.2022.02.004

[105] P. Bazard , J. Pineros , A. A. Acosta , M. Thivierge , L. R. Paganella , S. Zucker , F. L. Mannering , S. Modukuri , X. Zhu , R. D. Frisina , B. Ding , Hear Res. 2022, 426, 108625. 36215796 10.1016/j.heares.2022.108625

[106] H. Guo , K. Kunwar , D. Smith , J. Neurosci. 2017, 37, 9465. 28871035 10.1523/JNEUROSCI.1573-17.2017 PMC5618264

[107] L. Abuin , L. L. Prieto‐Godino , H. Pan , C. Gutierrez , L. Huang , R. Jin , R. Benton , BMC Biol. 2019, 17, 34. 30995910 10.1186/s12915-019-0651-7 PMC6472016

[108] M. A. Thompson , E. A. De‐Souza , Trends Cell Biol. 2024, 34, 176. 38008607 10.1016/j.tcb.2023.11.001

[109] L. Landskron , J. Bak , A. Adamopoulos , K. Kaplani , M. Moraiti , L. G. van den Hengel , J. Y. Song , O. B. Bleijerveld , J. Nieuwenhuis , T. Heidebrecht , L. Henneman , M.‐J. Moutin , M. Barisic , S. Taraviras , A. Perrakis , T. R. Brummelkamp , Science 2022, 376. 10.1126/science.abn6020 35482892

[110] K. Roy , E. P. Marin , Mol. Biol. Rep. 2018, 45, 1515. 30073588 10.1007/s11033-018-4224-6

[111] P. Tripathi , Z. Zhu , H. Qin , A. Elsherbini , S. M. Crivelli , E. Roush , G. Wang , S. D. Spassieva , E. Bieberich , J. Lipid Res. 2021, 62, 100021. 33380429 10.1194/jlr.RA120001190 PMC7903138

[112] B. Chen , J. Niu , J. Kreuzer , B. Zheng , G. K. Jarugumilli , W. Haas , X. Wu , Proc. Natl. Acad. Sci. U.S.A. 2018, 115. 10.1073/pnas.1800949115 PMC6130365 30127002

[113] M. Segal‐Salto , K. Hansson , T. Sapir , A. Kaplan , T. Levy , M. Schweizer , M. Frotscher , P. James , O. Reiner , Hum. Mol. Genet. 2017, 26, 1678. 28334871 10.1093/hmg/ddx074

[114] S. I. Motipally , S. Kolandaivelu , Adv. Exp. Med. Biol. 2023, 1415, 389. 37440062 10.1007/978-3-031-27681-1_57

[115] Z. S. Ulhaq , Y. Ogino , W. K. F. Tse , Biochem. Biophys. Res. Commun. 2023, 664, 100. 37141637 10.1016/j.bbrc.2023.04.096

[116] A. A. Kiseleva , V. A. Korobeynikov , A. S. Nikonova , P. Zhang , P. Makhov , A. Y. Deneka , M. B. Einarson , I. G. Serebriiskii , H. Liu , J. R. Peterson , E. A. Golemis , Clin. Cancer Res. 2019, 25, 4179. 30867219 10.1158/1078-0432.CCR-18-3535 PMC6606352

[117] D. F. McDermott , M. A. Huseni , M. B. Atkins , R. J. Motzer , B. I. Rini , B. Escudier , L. Fong , R. W. Joseph , S. K. Pal , J. A. Reeves , M. Sznol , J. Hainsworth , W. K. Rathmell , W. M. Stadler , T. Hutson , M. E. Gore , A. Ravaud , S. Bracarda , C. Suárez , R. Danielli , V. Gruenwald , T. K. Choueiri , D. Nickles , S. Jhunjhunwala , E. Piault‐Louis , A. Thobhani , J. Qiu , D. S. Chen , P. S. Hegde , C. Schiff , et al., Nat. Med. 2018, 24, 749. 29867230 10.1038/s41591-018-0053-3 PMC6721896

[118] N. O. Bukanov , L. A. Smith , K. W. Klinger , S. R. Ledbetter , O. Ibraghimov‐Beskrovnaya , Nature 2006, 444, 949. 17122773 10.1038/nature05348

[119] H. Husson , S. Moreno , L. A. Smith , M. M. Smith , R. J. Russo , R. Pitstick , M. Sergeev , S. R. Ledbetter , N. O. Bukanov , M. Lane , K. Zhang , K. Billot , G. Carlson , J. Shah , L. Meijer , D. R. Beier , O. Ibraghimov‐Beskrovnaya , Hum. Mol. Genet. 2016, 25, 2245. 27053712 10.1093/hmg/ddw093 PMC5081056

[120] C. L. Bonatto Paese , C. F. Chang , D. Kristeková , S. A. Brugmann , Dis. Model Mech. 2022, 15, 049611. 10.1242/dmm.049611 PMC9403750 35818799

[121] H. Mao , Z. Tang , H. Li , B. Sun , M. Tan , S. Fan , Y. Zhu , Y. Sun , Protein Cell 2019, 10, 726. 30850948 10.1007/s13238-019-0614-3 PMC6776484

[122] I.‐H. Lin , Y.‐R. Li , C.‐H. Chang , Y.‐W. Cheng , Y.‐T. Wang , Y.‐S. Tsai , P.‐Y. Lin , C.‐H. Kao , T.‐Y. Su , C.‐S. Hsu , C.‐Y. Tung , P.‐H. Hsu , O. Ayrault , B.‐C. Chung , J.‐W. Tsai , W.‐J. Wang , Cell Death Differ. 2024, 31, 1349. 38879724 10.1038/s41418-024-01325-2 PMC11445238

[123] L. Zhang , H. Zhang , E. Agborbesong , J. X. Zhou , X. Li , Cell Death Dis. 2023, 14, 795. 38052787 10.1038/s41419-023-06323-9 PMC10698143

[124] J. M. Tsai , R. P. Nowak , B. L. Ebert , E. S. Fischer , Nat. Rev. Mol. Cell Biol. 2024, 25, 740. 38684868 10.1038/s41580-024-00729-9

[125] R. N. Wang , L. Li , J. Zhou , J. Ran , Acta Pharmacol. Sin. 2025, 46, 805. 39775503 10.1038/s41401-024-01456-9 PMC11950361

[126] F. Yu , T. Li , Y. Sui , Q. Chen , S. Yang , J. Yang , R. Hong , D. Li , X. Yan , W. Zhao , X. Zhu , J. Zhou , Protein Cell 2020, 11, 852. 32607788 10.1007/s13238-020-00746-2 PMC7647980

[127] G. E. Jones , P. Ostergaard , A. T. Moore , F. C. Connell , D. Williams , O. Quarrell , A. F. Brady , I. Spier , F. Hazan , O. Moldovan , D. Wieczorek , B. Mikat , F. Petit , C. Coubes , R. A. Saul , G. Brice , K. Gordon , S. Jeffery , P. S. Mortimer , P. C. Vasudevan , S. Mansour , Eur. J. Hum. Genet. 2014, 22, 881. 24281367 10.1038/ejhg.2013.263 PMC3938398

[128] J. M. Robitaille , R. M. Gillett , M. A. LeBlanc , D. Gaston , M. Nightingale , M. P. Mackley , S. Parkash , J. Hathaway , A. Thomas , A. Ells , E. I. Traboulsi , E. Héon , M. Roy , S. Shalev , C. V. Fernandez , C. MacGillivray , K. Wallace , S. Fahiminiya , J. Majewski , C. R. McMaster , K. Bedard , JAMA Ophthalmol. 2014, 132, 1393. 25124931 10.1001/jamaophthalmol.2014.2814

[129] M. Liu , J. Ran , J. Zhou , Thorac. Cancer 2018, 9, 904. 29927078 10.1111/1759-7714.12792 PMC6068462

[130] J. Ran , H. Li , Y. Zhang , F. Yu , Y. Yang , C. Nie , S. Yang , D. Li , J. Zhou , M. Liu , Sci. Bull 2021, 66, 1620. 10.1016/j.scib.2021.02.001 36654295

[131] E. Colin , J. Daniel , A. Ziegler , J. Wakim , A. Scrivo , T. B. Haack , S. Khiati , A.‐S. Denommé , P. Amati‐Bonneau , M. Charif , V. Procaccio , P. Reynier , K. A. Aleck , L. D. Botto , C. L. Herper , C. S. Kaiser , R. Nabbout , S. N'Guyen , J. A. Mora‐Lorca , B. Assmann , S. Christ , T. Meitinger , T. M. Strom , H. Prokisch , The FREX Consortiumm , A. Miranda‐Vizuete , G. F. Hoffmann , G. Lenaers , P. Bomont , E. Liebau , Am. J. Hum. Genet. 2016, 99, 695. 27545681 10.1016/j.ajhg.2016.06.030 PMC5011045

[132] C. Erck , L. Peris , A. Andrieux , C. Meissirel , A. D. Gruber , M. Vernet , A. Schweitzer , Y. Saoudi , H. Pointu , C. Bosc , P. A. Salin , D. Job , J. Wehland , Proc. Natl. Acad. Sci. U.S.A. 2005, 102, 7853. 15899979 10.1073/pnas.0409626102 PMC1129054

[133] A. Chhatre , L. Stepanek , A. P. Nievergelt , G. Alvarez Viar , S. Diez , G. Pigino , Nat. Commun. 2025, 16. 10.1038/s41467-025-56098-0 PMC11770126 39865093

[134] L. M. Smith , N. L. Kelleher , Nat. Methods 2013, 10, 186. 23443629 10.1038/nmeth.2369 PMC4114032

[135] J. C. Tran , L. Zamdborg , D. R. Ahlf , J. E. Lee , A. D. Catherman , K. R. Durbin , J. D. Tipton , A. Vellaichamy , J. F. Kellie , M. Li , C. Wu , S. M. M. Sweet , B. P. Early , N. Siuti , R. D. LeDuc , P. D. Compton , P. M. Thomas , N. L. Kelleher , Nature 2011, 480, 254. 22037311 10.1038/nature10575 PMC3237778

[136] K. Jeanne Dit Fouque , S. A. Miller , K. Pham , N. V. Bhanu , Y. L. Cintron‐Diaz , D. Leyva , D. Kaplan , V. G. Voinov , M. E. Ridgeway , M. A. Park , B. A. Garcia , F. Fernandez‐Lima , Anal. Chem. 2022, 94, 15377. 36282112 10.1021/acs.analchem.2c03147 PMC11037235

[137] L. F. Schachner , K. Jooß , M. A. Morgan , A. Piunti , M. J. Meiners , J. O. Kafader , A. S. Lee , M. Iwanaszko , M. A. Cheek , J. M. Burg , S. A. Howard , M.‐C. Keogh , A. Shilatifard , N. L. Kelleher , Nat. Methods 2021, 18, 303. 33589837 10.1038/s41592-020-01052-9 PMC7954958

[138] M. Dupré , M. Duchateau , C. Malosse , D. Borges‐Lima , V. Calvaresi , I. Podglajen , D. Clermont , M. Rey , J. Chamot‐Rooke , J. Proteome Res. 2021, 20, 202. 32929970 10.1021/acs.jproteome.0c00351

[139] D. S. Roberts , J. A. Loo , Y. O. Tsybin , X. Liu , S. Wu , J. Chamot‐Rooke , J. N. Agar , L. Paša‐Tolić , L. M. Smith , Y. Ge , Nat. Rev. Methods Prim. 2024, 4, 38. 10.1038/s43586-024-00318-2 PMC11242913 39006170

[140] E. N. McCool , T. Xu , W. Chen , N. C. Beller , S. M. Nolan , A. B. Hummon , X. Liu , L. Sun , Sci. Adv. 2022, 8. 10.1126/sciadv.abq6348 PMC9770947 36542699

[141] L. M. Smith , J. N. Agar , J. Chamot‐Rooke , P. O. Danis , Y. Ge , J. A. Loo , L. Paša‐Tolić , Y. O. Tsybin , N. L. Kelleher , The Consortium for Top‐Down Proteomics , Sci. Adv. 2021, 7. 10.1126/sciadv.abk0734 PMC8589312 34767442

[142] Y. Cao , T. Yu , Z. Zhu , Y. Zhang , S. Sun , N. Li , C. Gu , Y. Yang , Pharmacol. Ther. 2024, 265, 108749. 39557344 10.1016/j.pharmthera.2024.108749

[143] H. Li , X. Sun , W. Cui , M. Xu , J. Dong , B. E. Ekundayo , D. Ni , Z. Rao , L. Guo , H. Stahlberg , S. Yuan , H. Vogel , Nat. Biotechnol. 2024, 42, 229. 38361054 10.1038/s41587-023-01987-2

[144] S. Mosalaganti , A. Obarska‐Kosinska , M. Siggel , R. Taniguchi , B. Turoňová , C. E. Zimmerli , K. Buczak , F. H. Schmidt , E. Margiotta , M.‐T. Mackmull , W. J. H. Hagen , G. Hummer , J. Kosinski , M. Beck , Science 2022, 376. 10.1126/science.abm9506 35679397

[145] J. Abramson , J. Adler , J. Dunger , R. Evans , T. Green , A. Pritzel , O. Ronneberger , L. Willmore , A. J. Ballard , J. Bambrick , S. W. Bodenstein , D. A. Evans , C.‐C. Hung , M. O'Neill , D. Reiman , K. Tunyasuvunakool , Z. Wu , A. Žemgulytė , E. Arvaniti , C. Beattie , O. Bertolli , A. Bridgland , A. Cherepanov , M. Congreve , A. I. Cowen‐Rivers , A. Cowie , M. Figurnov , F. B. Fuchs , H. Gladman , R. Jain , et al., Nature 2022, 630, 8016. 10.1038/s41586-024-07487-w PMC11168924 38718835

[146] K. A. High , M. G. Roncarolo , N Engl. J. Med. 2019, 381, 455. 31365802 10.1056/NEJMra1706910

[147] Q. Ling , J. A. Herstine , A. Bradbury , S. J. Gray , Nat. Rev. Drug Disc. 2023, 22, 789. 10.1038/s41573-023-00766-7 37658167

[148] Y. Yang , J. Ran , M. Liu , D. Li , Y. Li , X. Shi , D. Meng , J. Pan , G. Ou , R. Aneja , S.‐C. Sun , J. Zhou , Cell Res. 2014, 24, 1342. 25342559 10.1038/cr.2014.136 PMC4220159

[149] K. Ikegami , M. Setou , TTLL FEBS Lett. 2009, 8, 583. 10.1016/j.febslet.2009.05.003 19427864

